# DFT, Monte Carlo and molecular dynamics simulations for the prediction of corrosion inhibition efficiency of novel pyrazolylnucleosides on Cu(111) surface in acidic media

**DOI:** 10.1038/s41598-021-82927-5

**Published:** 2021-02-12

**Authors:** Rachid Oukhrib, Youness Abdellaoui, Avni Berisha, Hicham Abou Oualid, Jeton Halili, Kaltrina Jusufi, Mustapha Ait El Had, Hassan Bourzi, Souad El Issami, Fatmah Ali Asmary, Virinder S. Parmar, Christophe Len

**Affiliations:** 1 Apply Chemistry-Physic Team, Faculty of Sciences, Ibn Zohr University, Agadir, Morocco; 2 Faculty of Engineering, Environmental Engineering Department, Autonomous University of Yucatan, Mérida, Mexico; 3 Department of Chemistry, Faculty of Natural and Mathematics Science, University of Prishtina, 10000 Pristina, Kosovo; 4 Laboratory of Biotechnology, Materials and Environment, Faculty of Sciences, Ibn Zohr University, Agadir, Morocco; 5 Green Enenrgy Park, IRESEN, Ben Guerir, Morocco; 6 Laboratoire de Chimie Biomoléculaire, substances naturelles et Réactivité (URAC 16), Faculté des Sciences Semlalia, Université Cadi Ayyad, B.P. 2390, Marrakech, Morocco; 7 Laboratoire de Chimie Bioorganique et Macromoléculaire, Faculty of Sciences and Technics Marrakech (FSTMG), Université Cadi Ayyad, Marrakech, Morocco; 8 Chemistry Department, College of Science, King Saud University, Riyadh, 11451 Saudi Arabia; 9 Department of Chemistry and Environmental Science, Medgar Evers College, The City University of New York, 1638 Bedford Avenue, Brooklyn, NY 11225 USA; 10 Chimie ParisTech, PSL Research University, CNRS, Institute of Chemistry for Life and Health Sciences, 11 rue Pierre et Marie Curie, 75005 Paris, France

**Keywords:** Chemistry, Green chemistry, Sustainability

## Abstract

Five novel pyrazolylnucleosides have been evaluated theoretically for their corrosion inhibition efficiency on the Cu(111) surface in acidic media. DFT calculations were carried out to exhibit the intrinsic properties such as lowest unoccupied (E_LUMO_) and highest occupied (E_HOMO_) molecular orbital energies, as well as energy gap (∆E), chemical hardness (η), chemical softness (σ), electronegativity (χ), electrophilicity (ω) and nucleophilicity (ε). The theoretical FT-IR spectra were recorded to indicate the presence of the specific bonds in the studied molecules. The surface interactions between the inhibitor molecules and the metal surface were investigated using molecular dynamics simulations and *Monte Carlo* (MC) simulations. As a result, we have found that the inhibitor pyrazolylnucleosides **5a**–**e** have strong interactions with Cu(111) surface, and therefore have excellent predictive inhibition power against copper corrosion.

## Introduction

Copper corrosion is a serious challenge faced in the industry because of the broad applications of this metal and its alloys. Despite the corrosion resistance property of this metal in the atmosphere and some chemical environments, the pitting corrosion may occur on copper surface in the presence of oxygen and some aggressive anions, especially in acidic media^1^. However, this kind of corrosion is challenging and difficult to predict, detect, and protect against^2^. Therefore, and regarding the widespread use of copper in different industries, copper's corrosion protection issue has attracted much attention resulting in many conducted and ongoing studies^3^. The employment of corrosion inhibitors is the most efficient and less expensive approach to control the copper corrosion in acidic media. Chromates, molybdates, and tetraborates were the choice corrosion inhibitors for copper, but unfortunately, their use was accompanied by some challenges represented in toxicity, low efficiency and instability of the protective layer^4^. In contrast, organic adsorption inhibitors such as imidazolines and their derivatives were more efficient in protecting copper because of their high corrosion inhibition. The presence of heteroatoms like N, O, P, and S in these corrosion inhibitors' molecular structure serves as adsorption centers and facilitates adsorption on the copper surface. In recent years, some nucleoside-based molecules have been reported as a new class of corrosion inhibitors in acidic media^5–10^.

Nucleosides are the building blocks of nucleic acids consisting of nitrogen-rich heterocyclic linked sugar moieties via a *N*-glycosidic linkage. These platform molecules are of great importance to all living beings and determine the inherited features of every Life as they are considered subunits of nucleic acids^11^. However, most of the nucleoside analogs exhibit antiviral activities and also are shown to possess fungicidal and antitumor applications^12–14^. The art of modifications of nucleosides^5,6^ has gained great attention as a promising area of developing antiviral agents such as anti-HIV drugs, e.g. the heterocyclic ring replacement of the nucleoside sugar moieties has lead to a potent anti-HIV drug, 3TC^7,8^.

The combination of pyrazole and nucleoside moieties has been extensively explored to develop bioactive compounds against many diseases, but their side-effects still pose major problems in developing them for clinical uses. Indeed, the employment of pyrazolylnucleosides^14–16^ compounds in several applications has gained success by dint of their chemical and structural property. The novel pyrazolylnucleosides is pinning as an axis of further study and application in a relevant field to the metallic surface treatment to protect them against corrosion in a corrosive medium. The inhibition efficiency of pyrazolylnucleosides is undoubtedly due to the adsorption of their active sites, which are nitrogen and oxygen atoms. Otherwise, Several studies have shown that the novel pyrazolylnucleosides were devoid of any significant toxicity properties^17–21^.

In this work, five novel synthetic pyrazolylnucleosides, which showed potential anticancer activities have been theoretically studied for the first time as suitable effective corrosion inhibitors on copper surface Cu(111) in acidic medium^2,22–29^. In this regard, various theoretical methods such as density functional theory (DFT) calculations, dynamic molecular simulations (MD) and *Monte Carlo* (MC) techniques were carried out to study the intrinsic properties of the studied inhibitors to support this theoretical study^30–32^.

## Computational details

### DFT calculations

DFT calculations were conducted using the Dmol^3^ software (Biovia). Geometry optimization was completed via the use of the double numerical polarization basis set (DND)^33^ in combination with the M-11L functional within GGA^34–36^. Water was used as a solvent in DFT calculations in the Conductor-like Screening Model (COSMO)^37,38^.

### Monte Carlo simulations and molecular dynamics

For the Monte Carlo (MC) and Molecular dynamic (MD) simulations, the interaction of the copper surface and the inhibitor molecules in the simulated corrosion media was performed via the Cu(111) model (under Periodic Boundary Condition) employing the size of 30.672 Å × 30.672 Å × 8.477 Å with the inclusion of a 30 Å vacuum layer at C axis including 600 water molecules/one inhibitor molecule/15 hydronium + 15 chloride ions. MC calculations were realized by applying five cycles (2000 steps in each cycle) of simulated annealing. The lowest potential energy configurations were sampled at the steps of low temperatures. MD was obtained using an NVT canonical ensemble at 298 K and simulation time of 300 ps (1 fs time step)^39–43^. Temperature control was achieved via the use of the Berendsen thermostat 19. A frequently employed COMPASSII force field^44^ was used for the simulations^39,40,43,45,46^. For the computation of the Radial Distribution Function (RDF), the total trajectory was used^43,45,47,48^.

## Results and discussion

### DFT results

**Figure 1:**
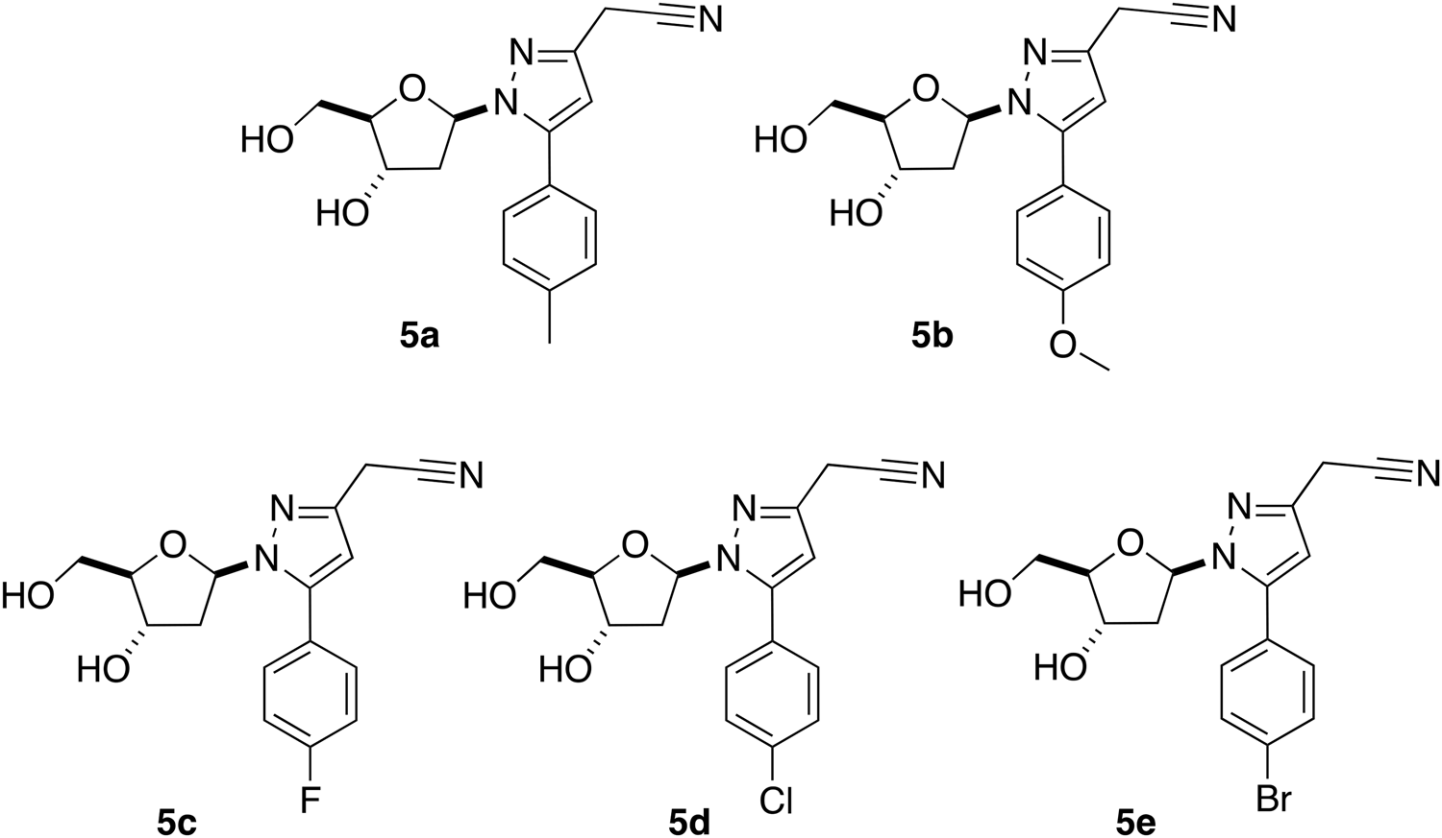
Structures of the pyrazolylnucleosides **5a**–**e**.

**Figure 2:**
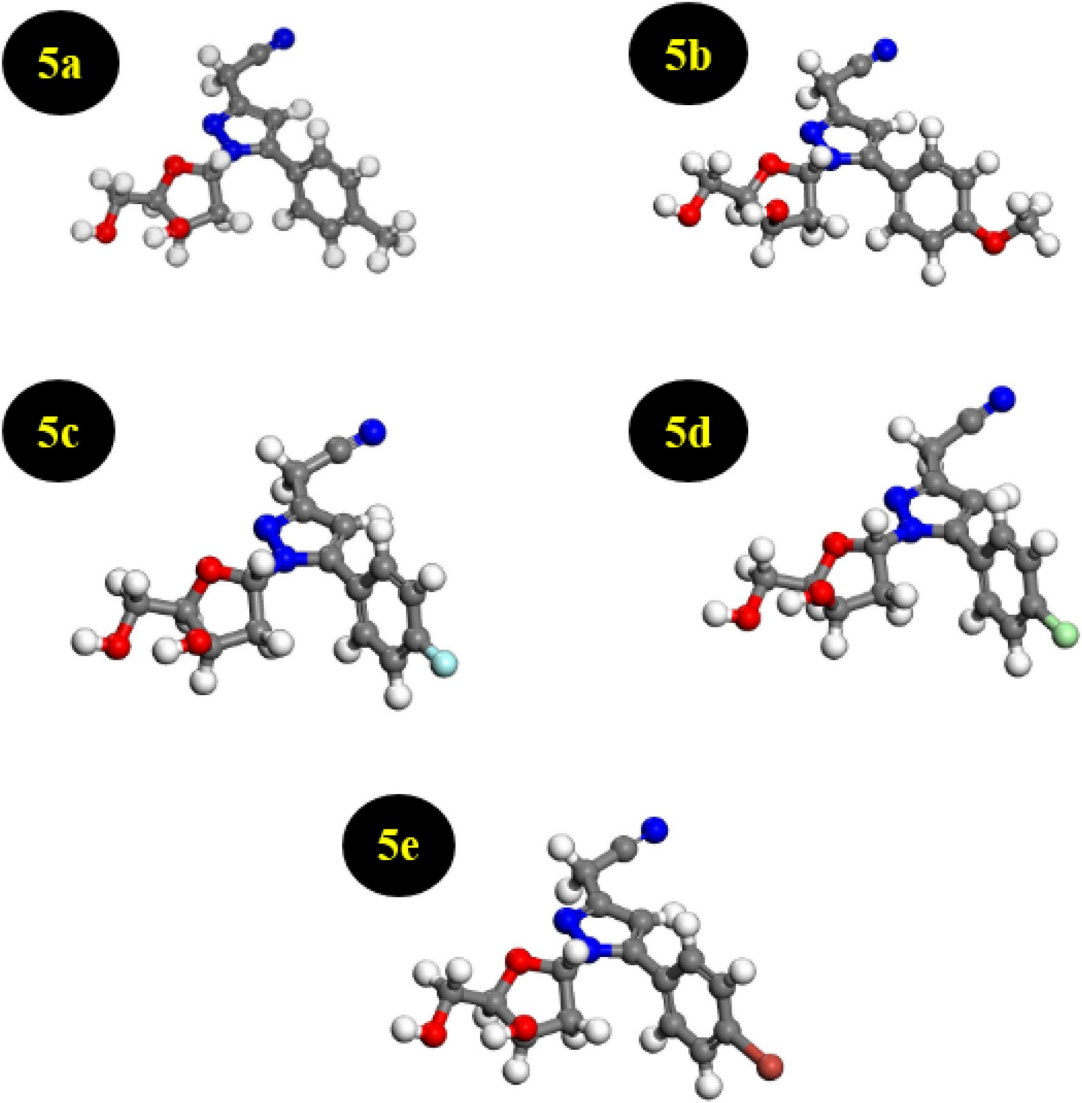
Optimized structures of the pyrazolylnucleosides **5a**–**e**.

The synthesis of the novel pyrazolylnucleosides **5a**–**e** used in the present study has been reported earlier by our Groups^17^. The inhibitory efficiency of the pyrazolylnucleosides **5a**–**e** (Fig. 1) has been studied by finding out the local parameters and quantifying the global parameters such as chemical hardness (η), softness (σ), electronegativity (χ), electrophilicity (ω) and nucleophilicity (ε). The protection abilities of these inhibitors have been evaluated based on the understanding of the distinctive and determining factors involved in their inhibitory capacities. The optimized structures of the pyrazolylnucleosides are presented in Fig. 2. Figure S1 illustrates the labeled model. Using density functional theory (DFT) calculations, these geometric structures were established using the Dmol^3^ software (Biovia). Optimization of the structure is a specific preliminary procedure that allows for a complete study of the reactivity of the inhibitors to interpret their effectiveness. In our approach, the optimized structures of the pyrazolylnucleosides **5a**–**e** were achieved by using the double numerical polarization basis set (DND) in combination with the M-11L functions within GGA.

Optimized structures were further subjected to the quantum chemical calculations in order to describe local properties such as the frontier molecular orbital (FMO) density distributions, i.e., the HOMO and the LUMO which, as presented in the discussion to follow, allowed us to determine the global parameters^49^. These studies collectively helped us to obtain insight into the inhibition mechanisms of these pyrazolylnucleosides towards the Cu(111) surface by examining the structure–reactivity correlation^49^.

**Figure 3:**
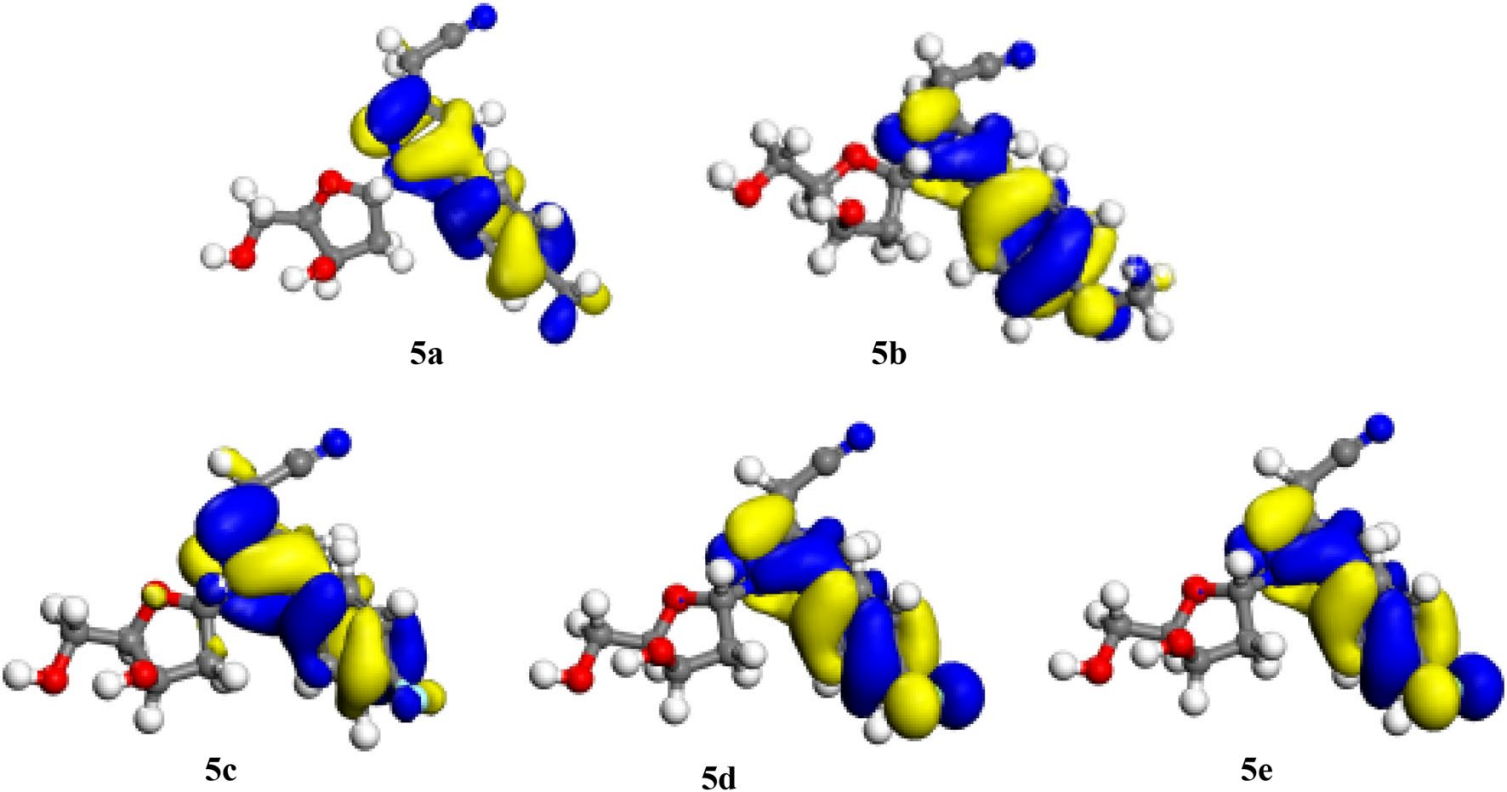
HOMO representations of the pyrazolylnucleosides **5a**–**e**.

**Figure 4:**
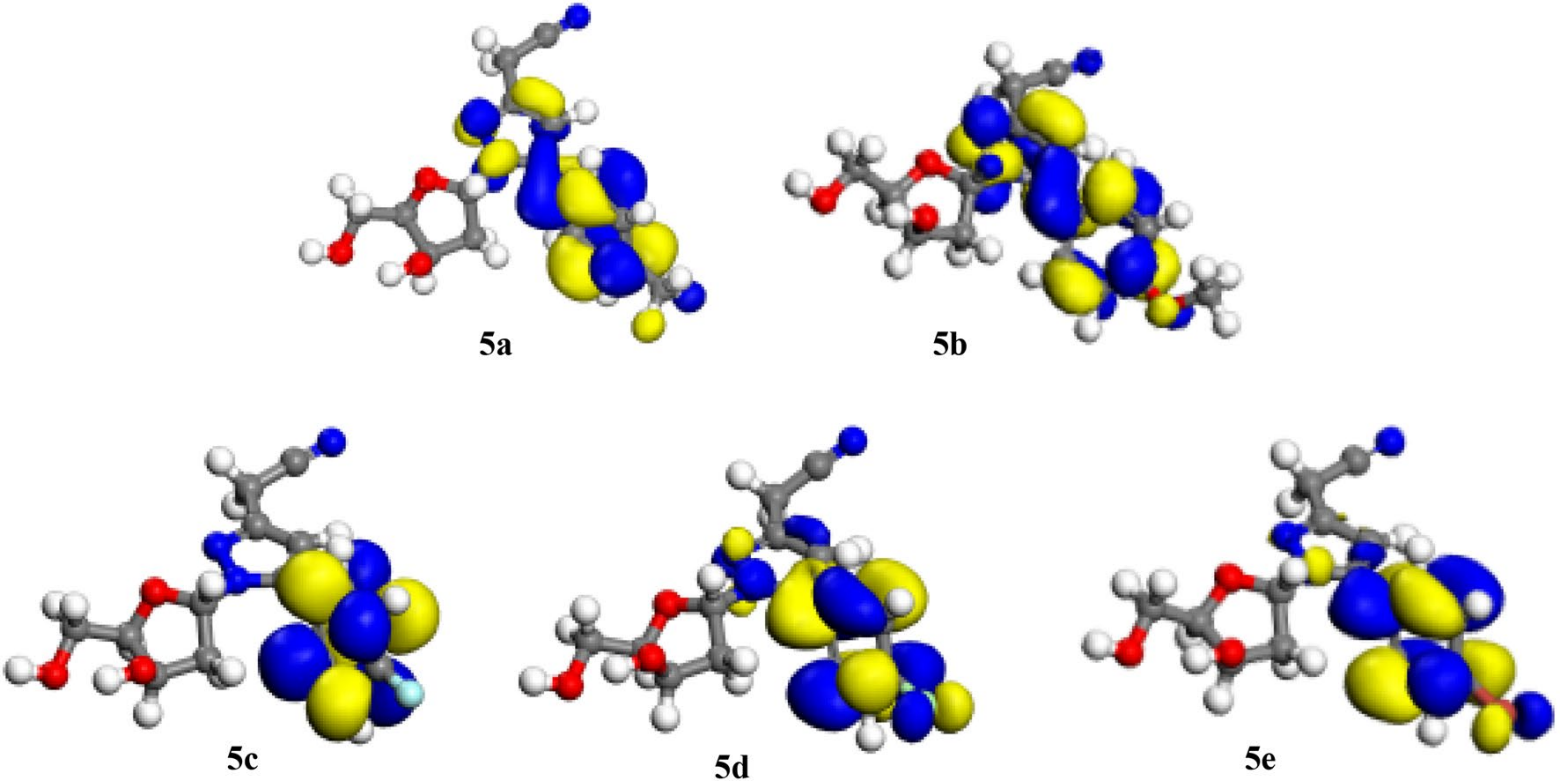
LUMO representations of the pyrazolylnucleosides **5a**–**e**.

Figures 3 and 4 show, respectively, the density distributions in frontier molecular orbitals, i.e. the HOMO and the LUMO; both HOMOs and LUMOs distributions are marked by inequality in all of the molecules. These results indicate that the pyrazolylnucleoside molecules possess active sites through which they can react with the metallic surface^50^. This finding could be explained by the fact that HOMOs are often associated with the ability to give electrons by inhibitor molecules to a suitable acceptor such as surface atoms to be protected from corrosion^51,52^, this could be explained later by the high values of E_HOMO_ energies which are indicative of the tendency of the molecule to donate an electron. Further, we have noticed that HOMOs and LUMOs distributions of these five pyrazolylnucleosides were concentrated preferentially within the structure around nitrogen (N) atoms and with a similar degree also around the oxygen (O) atoms despite their presence in several sites. This finding indicates that the reactions of these molecules with the surface Cu(111) will presumably take place through the parts of the pyrazolylcucleoside structure containing nitrogen and oxygen atoms such as [(N–N, C=N and C–N) and (C–O, O)]. These results have shed light on sites through which the inhibitors could interact with the studied surface^53,54^. Donor sites, as we reported previously, are the suitable sites for molecules preferentially bonded with positively polarized anodic reaction sites as with copper surface Cu(111) in acid media^55–57^, thereby reducing the migration of corrosive species onto the copper surface, which reflects a decreased rate of anodic copper dissolution reaction^58^. LUMO distribution depicts acceptor parts of the molecules^59^; this could explain the possibility of an interaction (adsorption) of the inhibitor molecules through its acceptor atoms on the metallic surface, often having a positive charge as mentioned previously^55–57^.

### Electrostatic potential maps (ESP)

**Figure 5:**
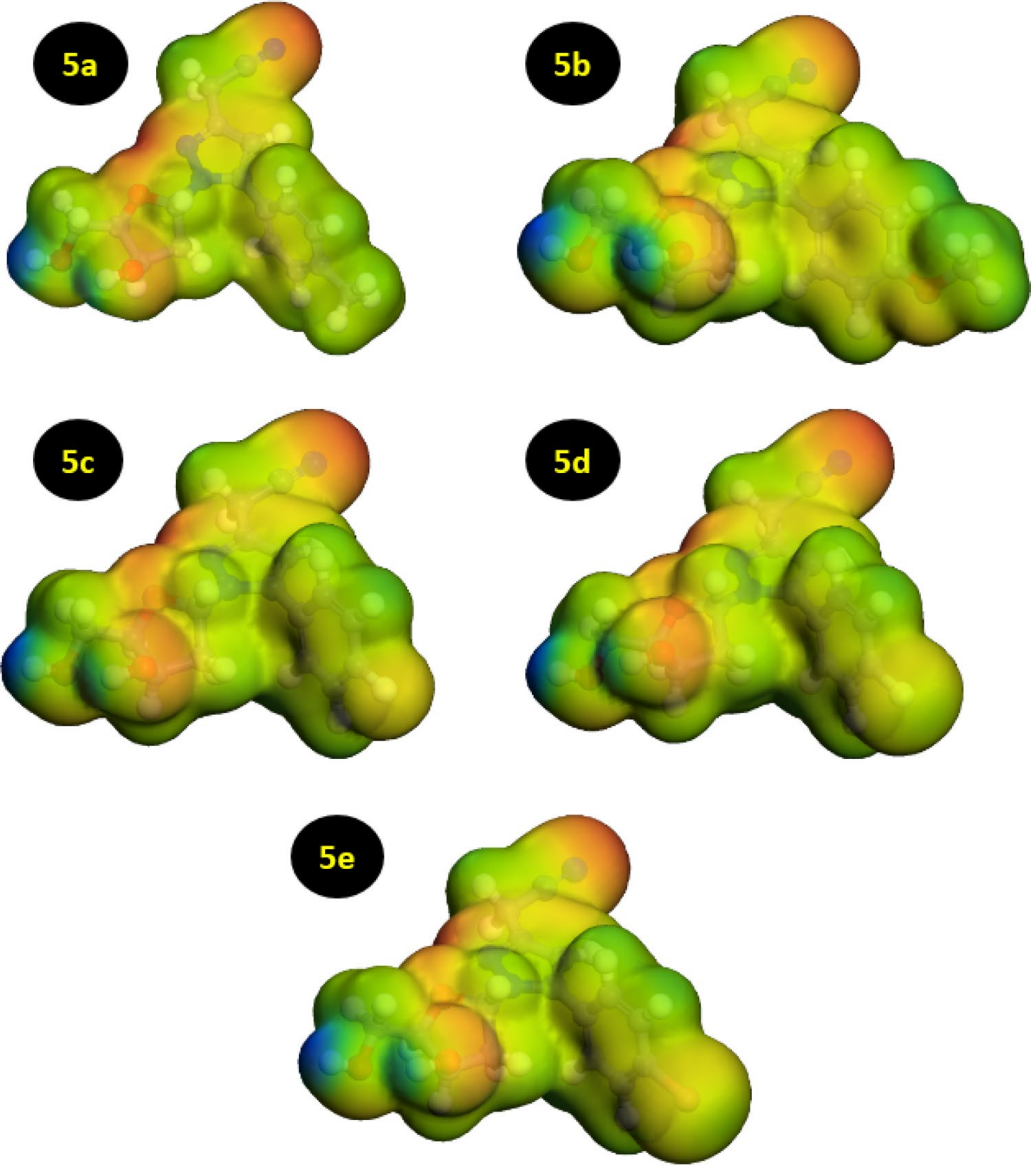
ESP graphic maps of the pyrazolylnucleosides **5a**–**e**.

By convention, the ESP map is related to nucleophilic and electrophilic activity sites in molecules; the red refers to the negative region while the green and blue ones refer to the positive region. As evidenced in Fig. 5, all of the red to yellow regions were distributed in negatively charged groups with heteroatoms, such as O, N atoms and around a few carbon atoms on their side or *O*-heterocyclic and *N*-heterocyclic rings^60,61^. The ESP maps reveal the reactive sites of inhibitors; in the case of pyrazolylnucleosides, oxygen and nitrogen atoms are shown to be the main adsorption sites. Therefore, it makes reasonable sense to consider that the pyrazolylnucleosides inhibitors contain several adsorption sites which are distinguished from each other predominantly by the N, O atoms.

### Mulliken charges

Table 1 lists the charges of C, Br, N, O, F, Cl atoms of the five studied pyrazolylnucleosides inhibitors. Many studies indicate a correlation between the corrosion inhibition efficiency of an inhibitor and its Mulliken charges^48,60,62–64^. It has been previously shown that atoms bearing most negative charges will share electrons more easily with the unoccupied orbital of the metal surface atoms with which they react^65^. Besides, such studies have indicated that the reactivity of these atom sites increases as the absolute value of charge density increases^66–68^. Consequently, the atoms that carry a pronounced negative charge in the pyrazolylnucleosides **5a**–**e** act probably as the active sites, through which these inhibitors adsorb onto the metallic surface Cu(111), the surface under studies in the present work. Indeed, the obtained results conclusively show that the negative charges concentrated on atoms like O, N, F, Br and Cl are the active sites in the five pyrazolylnucleosides as highlighted in Fig. 5 showing the charge distribution over the entire structures of the inhibitors under study.

**Table 1:** Mulliken atomic charges of the pyrazolylnucleosides **5a**–**e** inhibitors in their protonated forms.

5a		5b		5c		5d		5e	
Atoms	Charge	Atoms	Charge	Atoms	Charge	Atoms	Charge	Atoms	Charge
N (1)	**− 0.240**	N (1)	**− 0.268**	N (1)	**− 0.234**	N (1)	**− 0.261**	N (1)	**− 0.234**
C (2)	0.369	C (2)	0.383	C (2)	0.368	C (2)	0.383	C (2)	0.374
C (3)	**− 0.478**	C (3)	**− 0.488**	C (3)	**− 0.473**	C (3)	**− 0.478**	C (3)	**− 0.492**
C (4)	0.398	C (4)	0.407	C (4)	0.396	C (4)	0.413	C (4)	0.373
N (5)	**− 0.356**	N (5)	**− 0.353**	N (5)	**− 0.356**	N (5)	**− 0.365**	N (5)	**− 0.346**
C (6)	**− 0.758**	C (6)	**− 0.761**	C (6)	**− 0.757**	C (6)	**− 0.770**	C (6)	**− 0.763**
C (7)	0.491	C (7)	0.492	C (7)	0.488	C (7)	0.495	C (7)	0.433
N (8)	**− 0.520**	N (8)	**− 0.519**	N (8)	**− 0.516**	N (8)	**− 0.530**	N (8)	**− 0.507**
C (9)	0.080	C (9)	0.089	C (9)	0.072	C (9)	0.086	C (9)	0.074
C (10)	**− 0.297**	C (10)	**− 0.298**	C (10)	**− 0.290**	C (10)	**− 0.298**	C (10)	**− 0.320**
C (11)	**− 0.288**	C (11)	**− 0.336**	C (11)	**− 0.333**	C (11)	**− 0.261**	C (11)	**− 0.263**
C (12)	0.253	C (12)	0.613	C (12)	0.664	C (12)	0.138	C (12)	0.125
C (13)	**− 0.284**	C (13)	**− 0.362**	C (13)	**− 0.339**	C (13)	**− 0.267**	C (13)	**− 0.266**
C (14)	**− 0.295**	C (14)	**− 0.302**	C (14)	**− 0.284**	C (14)	**− 0.289**	C (14)	**− 0.314**
C (15)	**− 0.807**	O (15)	**− 0.675**	F (15)	**− 0.499**	Cl(15)	**− 0.131**	Br(15)	**− 0.179**
C (16)	0.305	C (16)	0.315	C (16)	0.297	C (16)	0.311	C (16)	0.277
O (17)	**− 0.670**	O (17)	**− 0.675**	O (17)	**− 0.663**	O (17)	**− 0.672**	O (17)	**− 0.656**
C (18)	0.124	C (18)	0.136	C (18)	0.116	C (18)	0.120	C (18)	0.095
C (19)	0.117	C (19)	0.099	C (19)	0.121	C (19)	0.110	C (19)	0.086
C (20)	**− 0.559**	C (20)	**− 0.558**	C (20)	**− 0.556**	C (20)	**− 0.580**	C (20)	**− 0.629**
C (21)	**− 0.118**	C (21)	**− 0.114**	C (21)	**− 0.120**	C (21)	**− 0.132**	C (21)	**− 0.178**
O (22)	**− 0.845**	O (22)	**− 0.836**	O (22)	**− 0.845**	O (22)	**− 0.849**	O (22)	**− 0.850**
O (23)	**− 0.846**	O (23)	**− 0.842**	O (23)	**− 0.845**	O (23)	**− 0.852**	O (23)	**− 0.855**
H (24)	0.223	H (24)	0.224	H (24)	0.225	H (24)	0.233	H (24)	0.257
H (25)	0.315	H (25)	0.316	H (25)	0.314	H (25)	0.323	H (25)	0.342
H (26)	0.314	H (26)	0.315	H (26)	0.314	H (26)	0.322	H (26)	0.342
H (27)	0.232	H (27)	0.234	H (27)	0.243	H (27)	0.250	H (27)	0.276
H (28)	0.226	H (28)	0.241	H (28)	0.258	H (28)	0.255	H (28)	0.283
H (29)	0.226	H (29)	0.251	H (29)	0.258	H (29)	0.256	H (29)	0.283
H (30)	0.224	H (30)	0.228	H (30)	0.237	H (30)	0.246	H (30)	0.274
H (31)	0.247	H (31)	0.228	H (31)	0.233	H (31)	0.236	H (31)	0.257
H (32)	0.247	H (32)	0.221	H (32)	0.223	H (32)	0.229	H (32)	0.250
H (33)	0.236	H (33)	0.226	H (33)	0.225	H (33)	0.232	H (33)	0.258
H (34)	0.230	H (34)	0.277	H (34)	0.276	H (34)	0.284	H (34)	0.303
H (35)	0.222	H (35)	0.269	H (35)	0.266	H (35)	0.274	H (35)	0.305
H (36)	0.225	H (36)	0.225	H (36)	0.228	H (36)	0.236	H (36)	0.257
H (37)	0.277	H (37)	0.215	H (37)	0.220	H (37)	0.226	H (37)	0.242
H (38)	0.267	H (38)	0.527	H (38)	0.535	H (38)	0.542	H (38)	0.547
H (39)	0.227	H (39)	0.528	H (39)	0.531	H (39)	0.534	H (39)	0.538
H (40)	0.220	C (40)	**− 0.385**						
H (41)	0.534	H (41)	0.231						
H (42)	0.531	H (42)	0.247						
		H (43)	0.233						

Bold values indicate the negative charges of main atoms that belong to the active regions which assisting in the corrosion inhibition

### Vibrational spectroscopy

**Figure 6:**
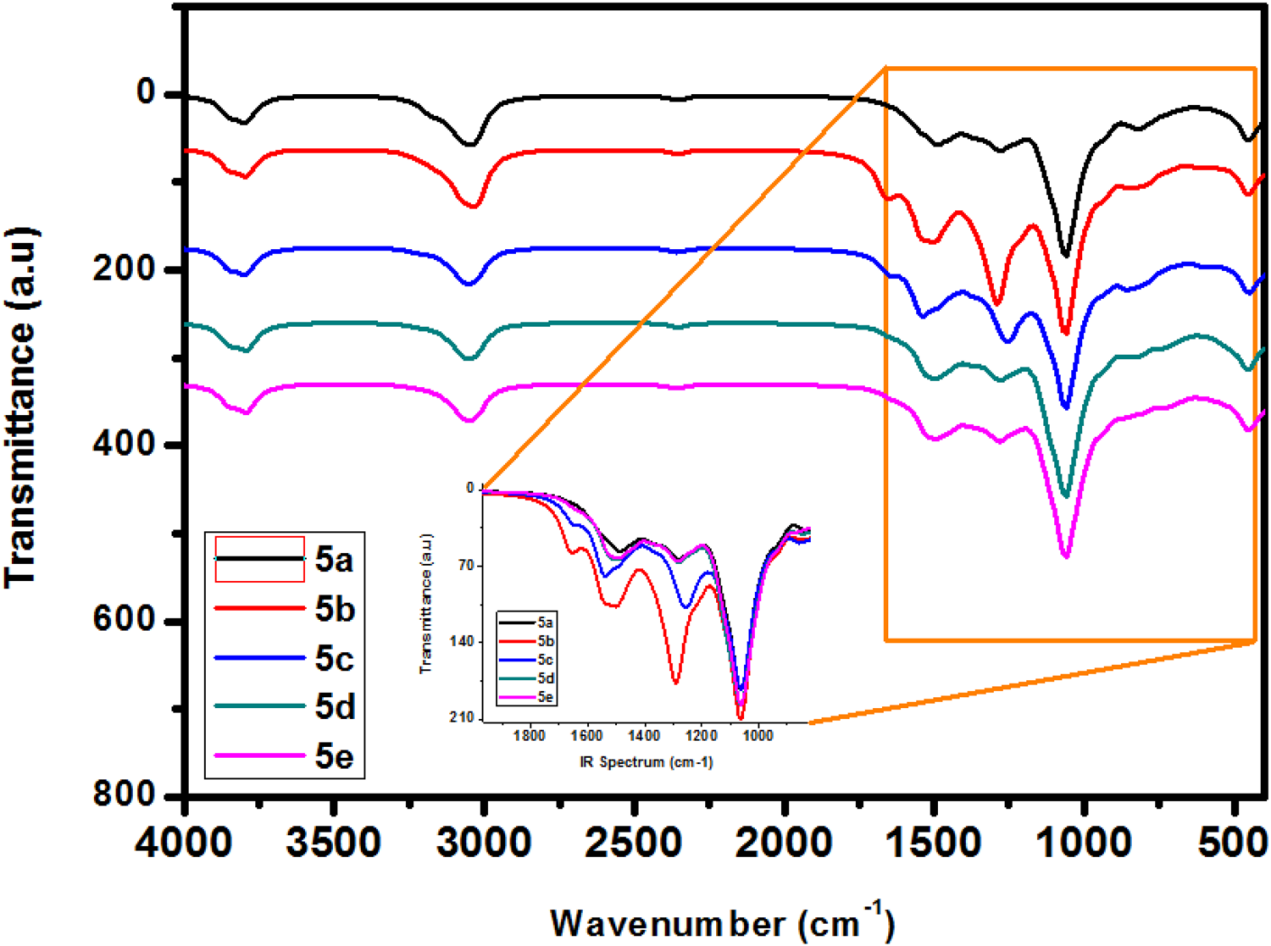
FTIR spectra of the pyrazolylnucleosides **5a**–**e**.

Figure 6 shows the vibrational spectra using an FT-IR spectrometer for the compounds **5a**–**e**. As shown in Fig. 6, the appearance of the characteristic bands of the base molecules was observed^69,70^ which confirmed their structures.

### Monte Carlo simulations

**Figure 7:**
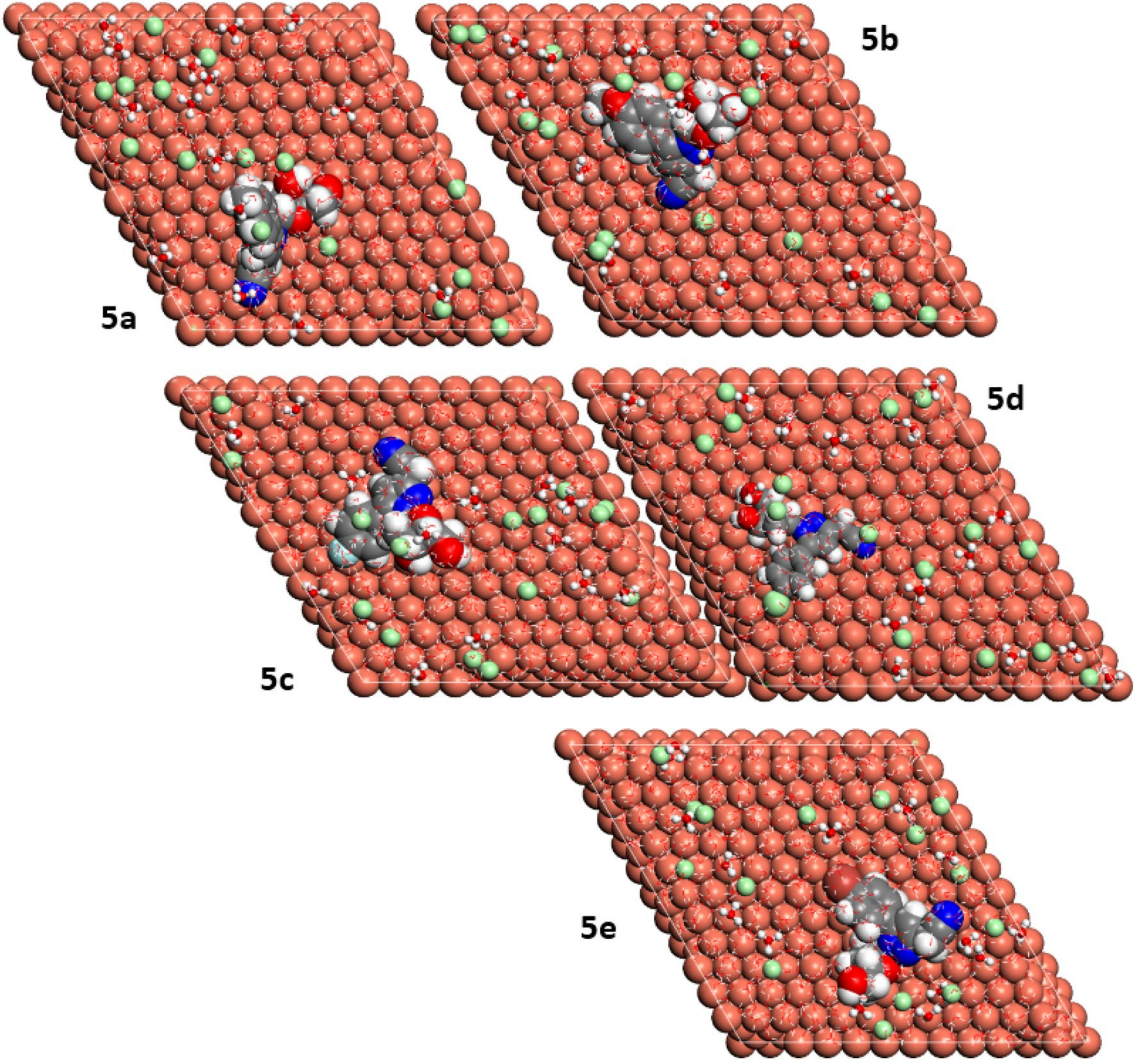
Top view of adsorption configurations of **5a**–**e** on Cu(111) in aqueous phase.

**Figure 8:**
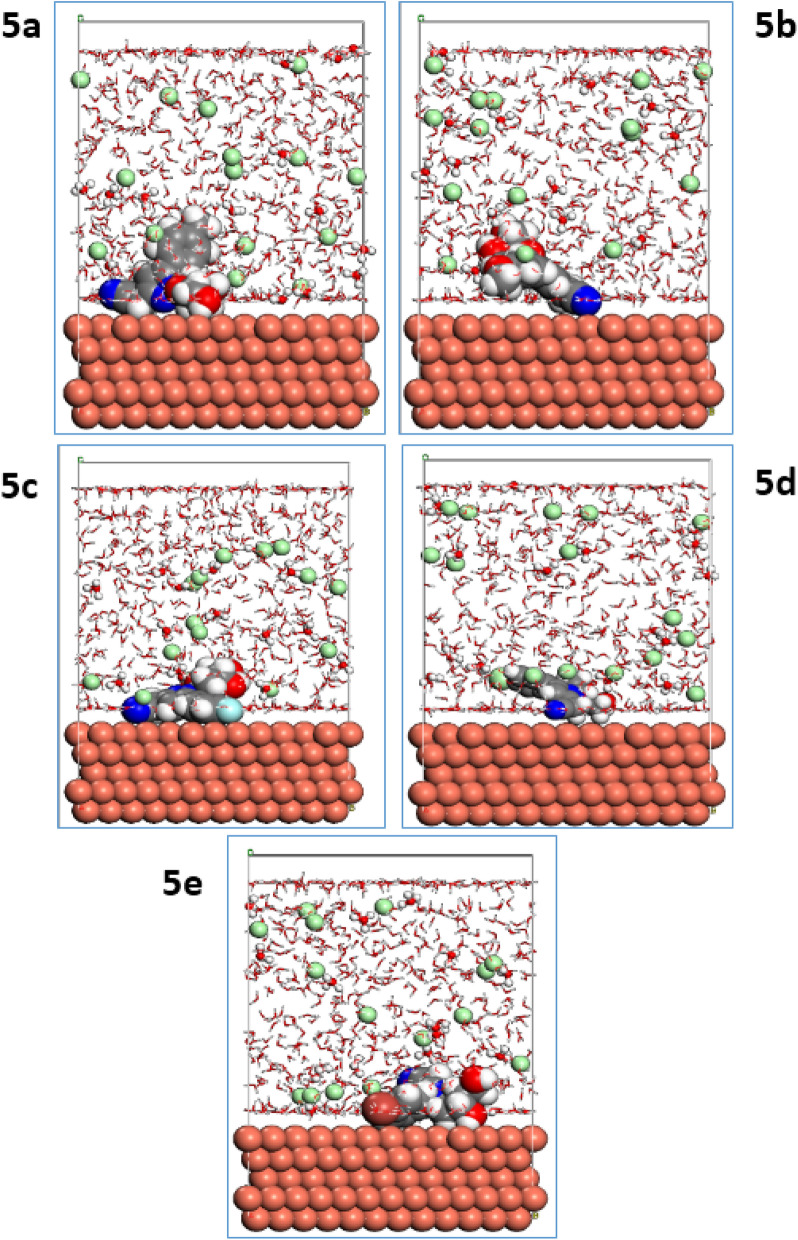
Side view of adsorption configurations of **5a**–**e** on Cu(111) in aqueous phase.

In the present study, the pyrazolylnucleosides **5a**–**e** are present in the protonated forms in the aqueous acidic media, the visual top and side surface configurations of the optimized inhibitors/Cu(111) are presented in Figs. 7 and 8, respectively. The closer positioning of the inhibitor molecules to the Cu(111) surface helps in the equilibrium adsorption configuration of the pyrazolylnucleosides to help them act as the corrosion inhibitors on the Cu(111) surface. It is clear from the side view pictures in Fig. 8 that all the pyrazolylnucleoside molecules are almost parallel to the Cu(111) surface, and all the five inhibitor molecules appear laying flat on the Cu(111) surface in the top view as seen in Fig. 7. Further, in the side view pictures shown in Fig. 8, the pyrazolylnucleosides **5a**, **5c** and **5e** look parallel to the Cu(111) surface and the pyrazolylnucleosides **5b** and **5d** are seen as quasi-parallel to the metal surface. This may be due to the differences between the sizes and the extents of their −I or +I effects of the five substituents (CH_3_, OCH_3_, F, Cl and Br)^71–74^. These MC simulations, as seen in the later discussion are quite useful in understanding the detailed mechanism of adsorption behavior of these inhibitors on the Cu(111) surface.

**Figure 9:**
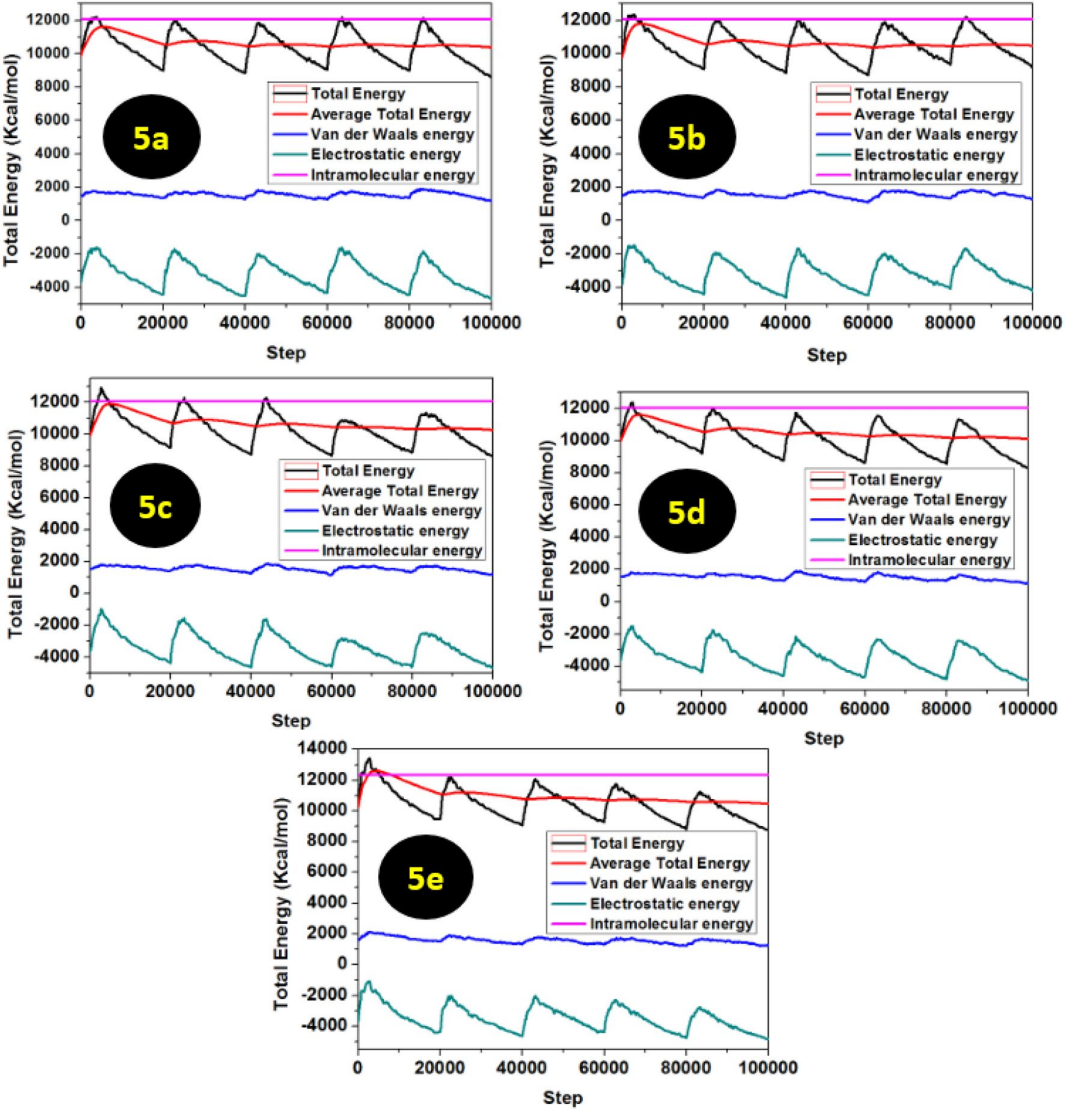
Energy fluctuation curves obtained from *MC* simulations for **5a**–**e**.

In order to demonstrate and confirm the equilibration of the systems, the correlation between the stable mean values of temperature and energy fluctuation was studied^43^. Figure 9 shows thermal fluctuations of the pyrazolylnucleosides **5a**–**e**, according to simulation time.

### Molecular dynamics (MD) calculations

**Figure 10:**
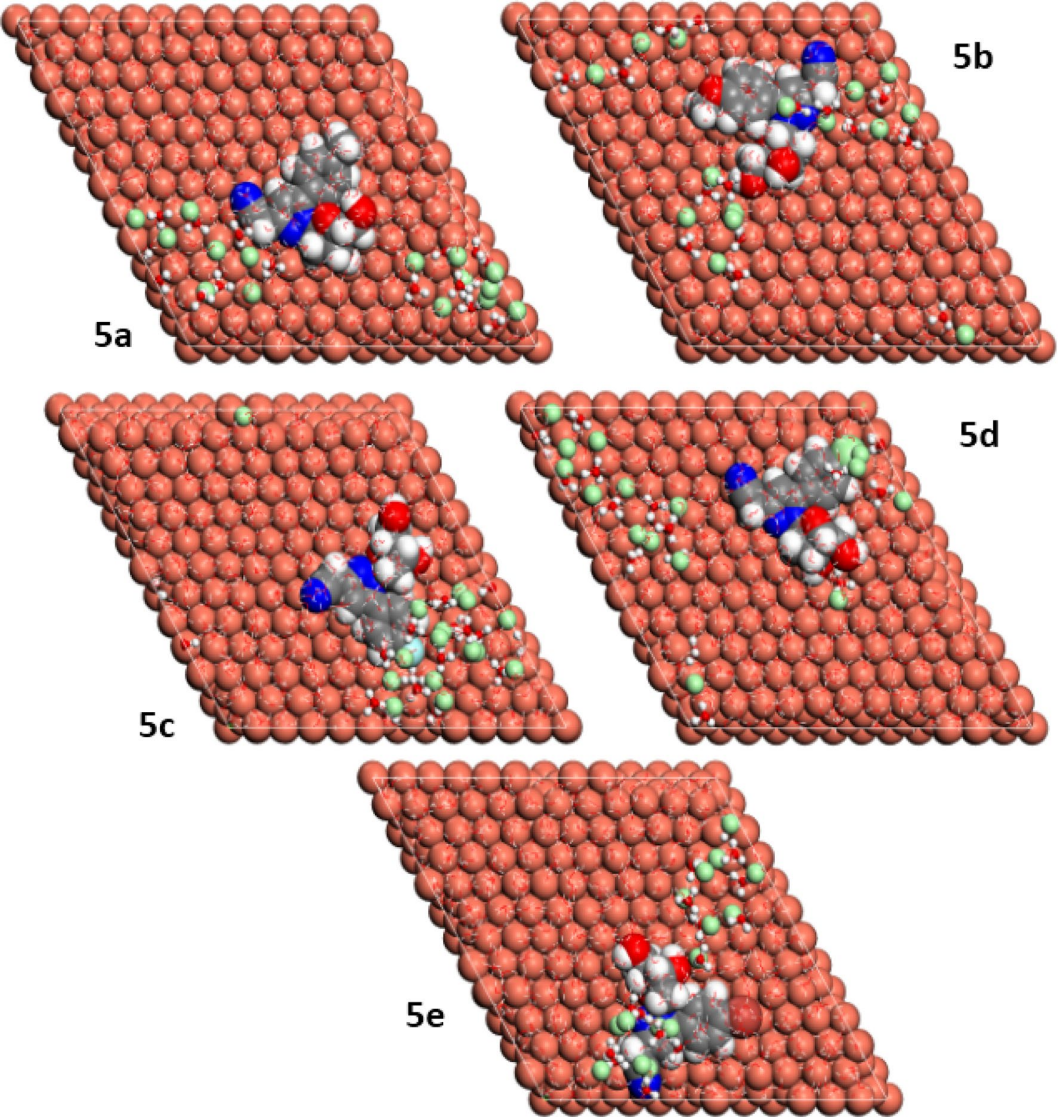
Lowest energy MD top view of **5a**–**e** onto Cu(111) surface.

**Figure 11:**
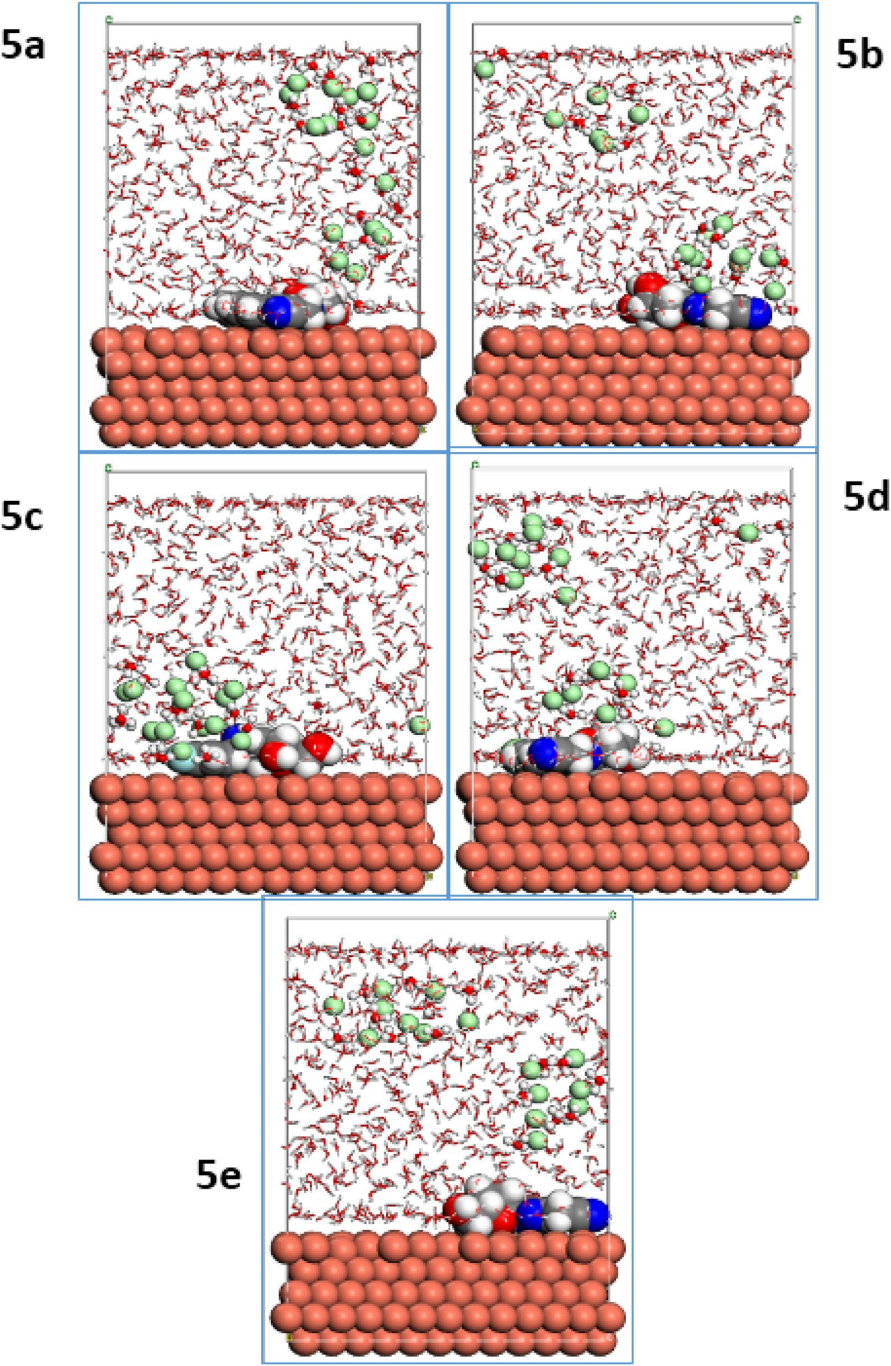
Lowest energy MD side view of **5a**–**e** onto Cu(111) surface.

To explain the interactions between the studied surface of copper and the active sites of the pyrazolylnucleosides **5a**–**e**, we launched the MD simulations in a system containing 600 water molecules and one molecule of each of the five inhibitors on the Cu(111) surface^43,75^; the visual simulations showed the corresponding adsorption mechanism of the pyrazolylnucleoside derivatives on the copper surface to gain a deeper understanding of the interaction between each inhibitor and Cu(111) surface. The results presented in Figs. 10 and 11 show, respectively, the top and side views of the realistic simulations^76,77^ of the pyrazolylnucleosides **5a**–**e** on the studied copper surface Cu(111) at equilibrium in the aqueous phase. All of the novel five inhibitors **5a**–**e** adsorb tightly onto the copper surface with a parallel orientation, more particularly the inhibitor **5e,** which appears close and parallel with the Cu(111) surface. It is entirely consistent with the previous results shown earlier by Monte Carlo simulations. This positioning is facilitated by the formation of bonds between the inhibitor and copper surface, formed through the sharing of p electrons from the active donor sites of the inhibitor pyrazolylnucleosides to the vacant orbitals of the positively charged copper surface^78^.

The adsorption of the inhibitor by adopting parallel placement of the inhibitor molecule and the Cu(111) surface explains how an inhibitor can minimize the contact area between corrosive elements and surface of Cu(111) in a corrosive environment^79^, in addition to the distinction between studied inhibitors according to the predictive efficacy. The different energies of the studied inhibitor molecules **5a**–**e** and Cu(111) substrate have been calculated and are summarized in the Tables S1, S2, S3, S4 and S5 (supporting informations). We have taken into account other corrosive species such as H_3_O^+^, Cl^−^ and H_2_O present in the environment in these calculations^80,81^. The output data determined via this simulation method gives the total energy (symbolized as E_tot_), which equals the sum of the internal and the adsorption energies of the inhibitor as an adsorbate on the metallic surface^82^. The total energy can be envisaged to correlate the reactivity of the inhibitor and we noticed that the pyrazolylnucleosides **5a**–**e** are quite stable since their energies are small, not exceeding − 7.21 E^**+03**^. The average of all the total energies calculated was found to be − 7.23 E^**+03**^. Adsorption energy (E_ads_) is the energy released when an inhibitor molecule (adsorbate) attaches to the metal surface Cu(111) (substrate), and includes the rigid adsorption energy and the deformation energy^83^. The adsorption energy refers to the energy released during the adsorption of the inhibitor on the Cu(111) surface in its stable state (also called the geometric optimization step), and the deformation energy is that released when the adsorbed inhibitor is released from the Cu(111) surface. It can be seen from the results tabulated in Tables S1, S2, S3, S4 and S5 that the pyrazolylnucleosides **5a**–**e** adsorb spontaneously on the Cu(111) surface as the values of the adsorption energies are negative^84,85^. The results also show that despite the presence of the corrosive elements such as H_2_O, H_3_O^+^ and Cl^−^, the inhibitors **5a**–**e** adsorb preferentially on the Cu(111) surface without significant competition because the adsorption of the pyrazolylnucleosides requires less energy. The adsorption energy (E_ads_) of the pyrazolylnucleoside **5e** was − 1.96 × 10^4^, while the average E_ads_ values of the other four pyrazolylnucleosides **5a**–**d** were − 1.93 × 10^4^. Also the desorption energy (dE_ads_/dN_i_) for the pyrazolylnucleoside **5e** was the lowest viz. − 438.8210 as compared to those of − 147.9912 for **5b**, − 146.7689 for **5a**, − 144.7019 for **5c** and − 137.7662 for **5d**. This indicates that **5e** requires slightly less energy for adhesion (adsorption) to the Cu(111) surface compared to the other inhibitors **5a**–**d**, which require more energy (E_ads_) for adsorption. On the other hand, the pyrazolylnucleoside **5e** can be released from the Cu(111) surface requiring much less desorption energy (dE_ads_/dN_i_) as compared to the other four inhibitors **5a**–**d** that need more desorption energy. From this study, an important result could be drawn viz. it is not possible to predict the inhibitory power of a molecule accurately from its adsorption energy alone; other elements also contribute in determining the effectiveness of an inhibitor, one of them being the desorption energy (dE_ads_/dN_i_). These results can help in picking efficient inhibitors against corrosion of any metallic surface in a corrosive environment combining the two factors viz. the adsorption energy and the desorption energy. Also, one can predict the inhibitory efficacy of the inhibitors as these studies help in classifying them according to their effectiveness against corrosion of Cu(111) surface; we conclude from these results that the effectiveness of the pyrazolylnucleoside inhibitors under study follows the order: **5e > 5b > 5a > 5c > 5d**.

**Figure 12:**
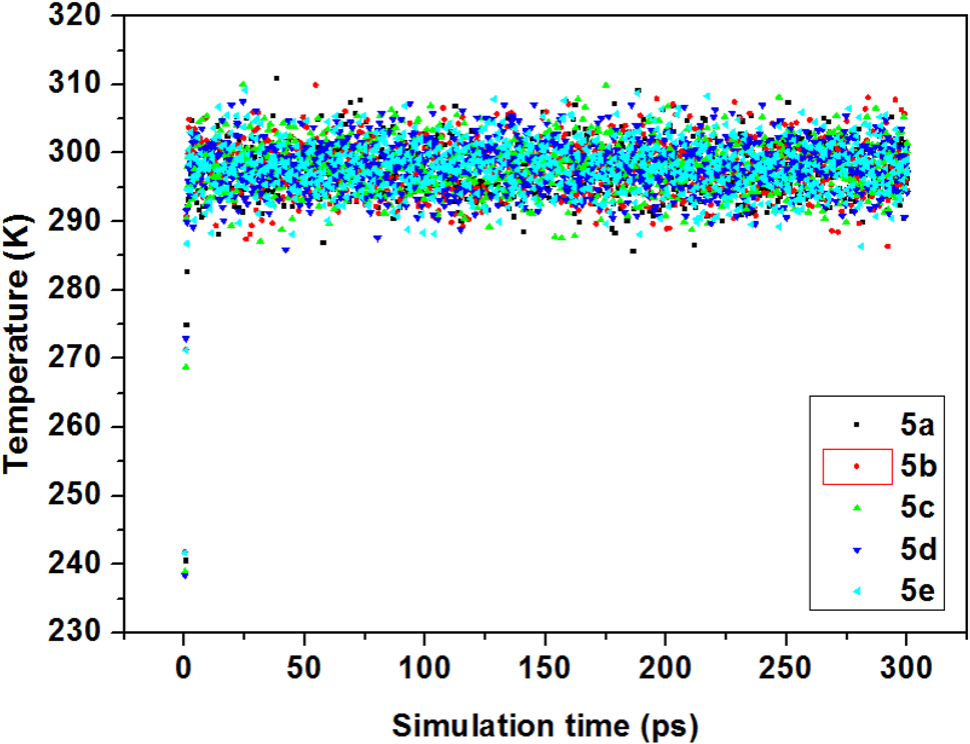
Temperature equilibrium curves obtained from MD simulations for **5a**–**e**.

To further confirm our results, we have performed the energy fluctuation curves as obtained from MD simulations; the equilibration of the system is confirmed by the stable mean values of energy fluctuations, as shown in Fig. 12.

**Figure 13:**
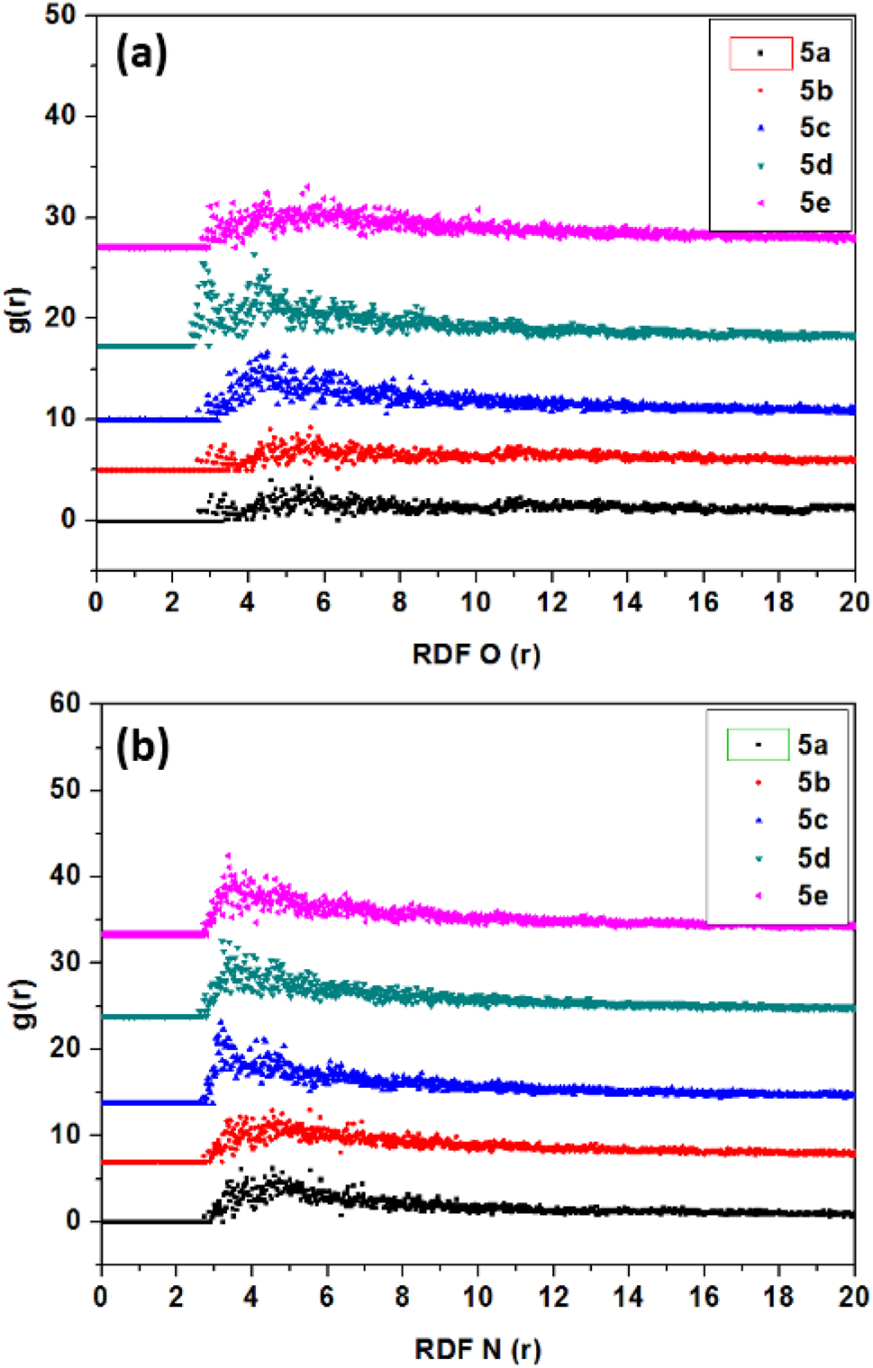
RDF O (**a**) and RDF N (**b**) of the pyrazolylnucleosides **5a**–**e** on the Cu(111) surface in solution.

The pair correlation function quantifies how other particles surround the particle of interest (or the targeted atom); based on this, we have used the radial distribution function (RDF) to estimate the length of the bond g(r)^86–88^. Knowing that the peak between 1 and 3.5 Å corresponds to chemisorption and that physisorption is associated with peaks greater than 3.5Å24, we have carried out RDF calculations as shown in Fig. 13a,b, respectively, for the RDF O and RDF N. The optimal short distances between the probable active sites of the studied inhibitors **5a**–**e** and the copper(111) surface atoms were: length of the bond of Cu\\O (2.85 to 3.36 Å) and Cu\\N (3.16 to 3.40 Å) which are less than 3.5 Å. The obtained RDF results show that the five pyrazolylnucleoside inhibitors have a great capacity to adsorb on Cu(111) surface, and subsequently protect it from dissolution^89,90^.

### Mechanism of adsorption and inhibition

**Figure 14:**
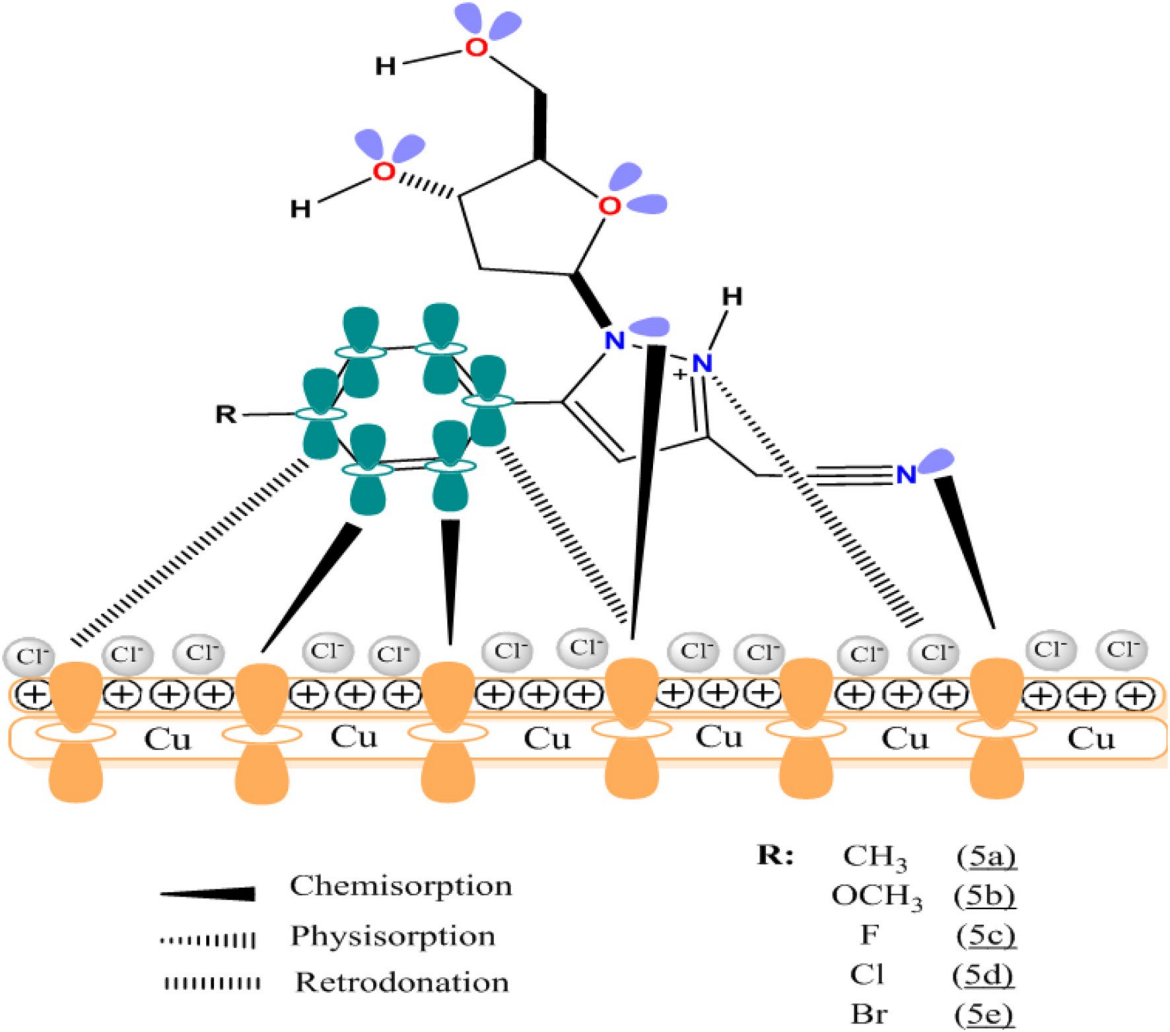
Schematic illustration of the adsorption mechanism of organic corrosion inhibitors **5a**–**e** on the surface of copper in a 1 M HCl solution.

We have worked out a simple approach to explain the reaction of the inhibitor molecules **5a**–**e** and the surface of copper, which is positively charged; interactions of organic inhibitors with metal surfaces involve donor–acceptor interactions^91–93^. Figure 14 illustrates the adsorption mechanism of the organic corrosion inhibitors **5a**–**e** on the copper metal surface in the acidic medium (1 M HCl). The metallic element Cu undergoes rapid oxidation due to the aggressive environment that makes the metal surface positively charged, which in turn favors the fixation of negatively charged counter chloride ions resulting in a negative metallic surface. As shown in Fig. 14, neutral nitrogen atoms of the five-membered pyrazole rings of the inhibitor molecules **5a**–**e** get protonated in the acidic solution according to the following reaction:

$$\left[ {{\text{Inh}}} \right]\, + \,{\text{xH}}^{ + } \leftrightarrow \left[ {{\text{Inh}}_{{\text{x}}} } \right]^{{{\text{x}} + }}$$

The protonated inhibitor molecules bind to the negatively charged metal surface through attractive electrostatic forces. In parallel, the lone pair electrons of the –CN moiety, the non-protonated pyrazole ring nitrogen atom and the oxygen atom of the sugar moiety of the inhibitor molecules, as well as the π-electrons of the benzene ring, could supply electrons to the vacant d-orbitals of the Cu atoms which leads to the phenomenon of chemisorption and retro-donation, respectively^94,95^. However, this type of electron transfer causes electrons to accumulate in the d-orbitals of the metal atoms resulting in inter-electron repulsions. In order to avoid this repulsion phenomenon, a reverse transfer of electrons takes place from the d-orbitals of the surface metal atoms to the unoccupied molecular orbitals of the inhibitor molecules (retro-donation), thus reinforcing the adsorption of the inhibitor molecules on the metal surface.

Consequently, it can be expected that the adsorption of different inhibitor molecules on the metal surface of copper in an aggressive acidic solution (1 M HCl) happens by three types of phenomena: physisorption, chemisorption and retro-donation. Furthermore, theoretical studies presented here show a good correlation with electrochemical studies, which show that these inhibitors have high metal corrosion inhibition performance. The presence of electron-donating mesomeric substituents –OCH_3_, Cl, Br and F further enhances the inhibitory efficacy of the pyrazolylnucleosides **5a**–**e** against copper corrosion.

## Conclusion

The inhibitory effects of five novel pyrazolylnucleosides have been evaluated theoretically against corrosion of copper surface in an acidic environment. Density functional theory (DFT) calculations were carried out to exhibit their intrinsic properties and reactivities. We used molecular dynamics simulation to describe the different probable interactions such as van der Waals and electrostatic interactions between the inhibitors **5a**–**e** and Cu(111) surface. The following conclusions can be drawn from the results:

1. Molecular dynamic simulations show that the inhibitor pyrazolylnucleosides **5a**–**e** have strong interactions with Cu(111) surface.
2. Molecular quantum chemical calculations showed that the reactive sites in the inhibitors **5a**–**e** are mainly the N-atoms and O-atoms.
3. Based on the analysis of the different outputs, we have suggested a probable reaction mechanism for the binding of the pyrazolylnucleosides **5a**–**e** at the Cu(111) surface.
4. A combination of adsorption energy (E_ads_) and the desorption energy (dE_ads_/dN_i_) values helps in determining the effectiveness of the pyrazolylnucleosides **5a**–**e** against corrosion of Cu(111) surface.
5. Molecular dynamic simulations reveal that the effectiveness of these inhibitors follows the order: **5e > 5b > 5a > 5c > 5d**.

## Supplementary Information

Supplementary Information.
