# Supplementary material for: DFT, Monte Carlo and molecular dynamics simulations for the prediction of corrosion inhibition efficiency of novel pyrazolylnucleosides on Cu(111) surface in acidic media
Source: Sci Rep. 2021 Feb 12;11:3771. doi: 10.1038/s41598-021-82927-5 (PMC7881149; doi:10.1038/s41598-021-82927-5)
Supplement: Supplementary file 1 — Supplementary Information. [file 41598_2021_82927_MOESM1_ESM.docx]

**DFT, Monte Carlo and molecular dynamics simulations for the prediction of corrosion inhibition efficiency of novel pyrazolylnucleosides on Cu(111) surface in acidic media**

Rachid Oukhrib^1^, Youness Abdellaoui^2^, Avni Berisha^3^, Hicham Abou Oualid^4,5^, Jeton Halili^3^, Kaltrina Jusufi^3^, Mustapha Ait El Had^6,7^, Hassan Bourzi^1^, Souad El Issami^1^, Fatmah Ali Asmary^8^, Virinder S. Parmar^9^ and Christophe Len^10*^

^1^ *Apply Chemistry-Physic Team, Faculty of Sciences, Ibn Zohr University, Agadir, Morocco.*

^2^ *Faculty of Engineering, Environmental Engineering Department, Autonomous University of Yucatan, Mérida, Mexico.*

^3^*Department of Chemistry, Faculty of Natural and Mathematics Science, University of Prishtina, 10000, Prishtina, Kosovo*

^4^ *Laboratory of Biotechnology, Materials and Environment, Faculty of Sciences, Ibn Zohr University, Agadir, Morocco.*

^5^ *Green Enenrgy Park, IRESEN, Benguerir.*

^6^*Laboratoire de Chimie Biomoléculaire, substances naturelles et Réactivité (URAC 16), Faculté des Sciences Semlalia, Université Cadi Ayyad, B.P. 2390, Marrakech, Morocco.*

^7^*Laboratoire de Chimie Bioorganique et Macromoléculaire, Faculty of Sciences and  Technics Marrakech (FSTMG), Université Cadi Ayyad, Marrakech, Morocco*

^8^*Chemistry Department, College of Science, King Saud University, Riyadh 11451, Saudi Arabia*

*^9^Department of Chemistry and Environmental Science, Medgar Evers College, The City University of*

*New York, 1638 Bedford Avenue, Brooklyn, NY 11225, USA.*

^10^*Chimie ParisTech, PSL Research University, CNRS, Institute of Chemistry for Life and Health Sciences, 11 rue Pierre et Marie Curie, F-75005 Paris, France.*

** Correspondence:* [christophe.len@chimieparistech.psl.eu](mailto:christophe.len@chimieparistech.psl.eu) (C.L.)

| **5a** | **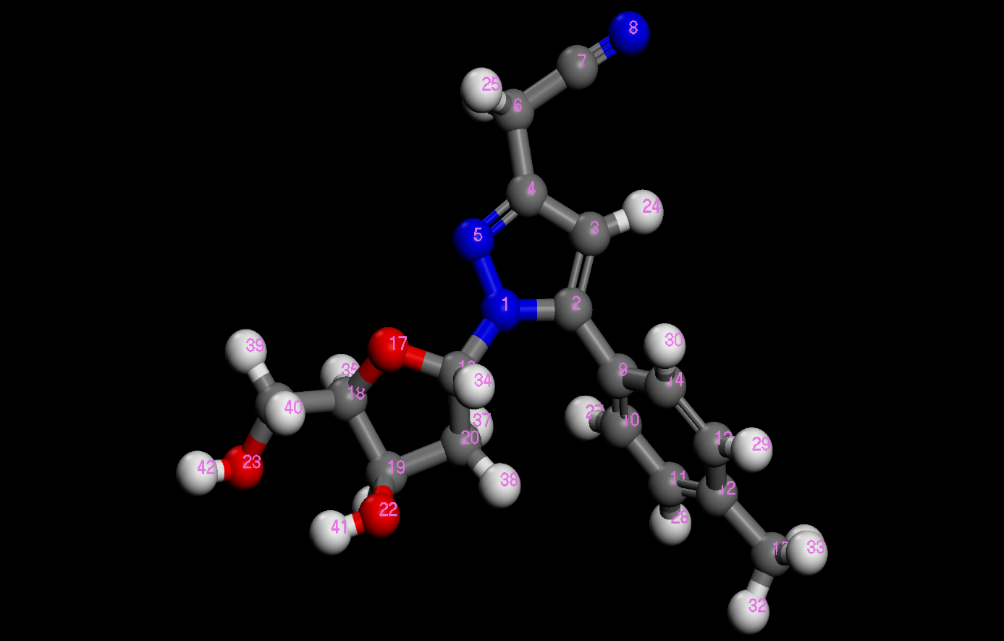** | **5b** | **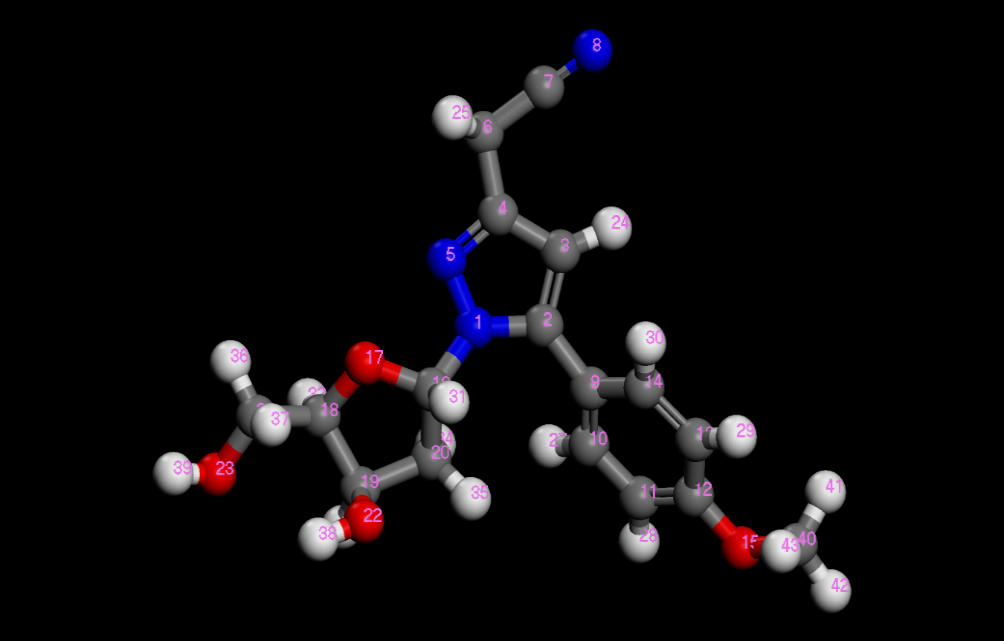** |
| --- | --- | --- | --- |
| **5c** | **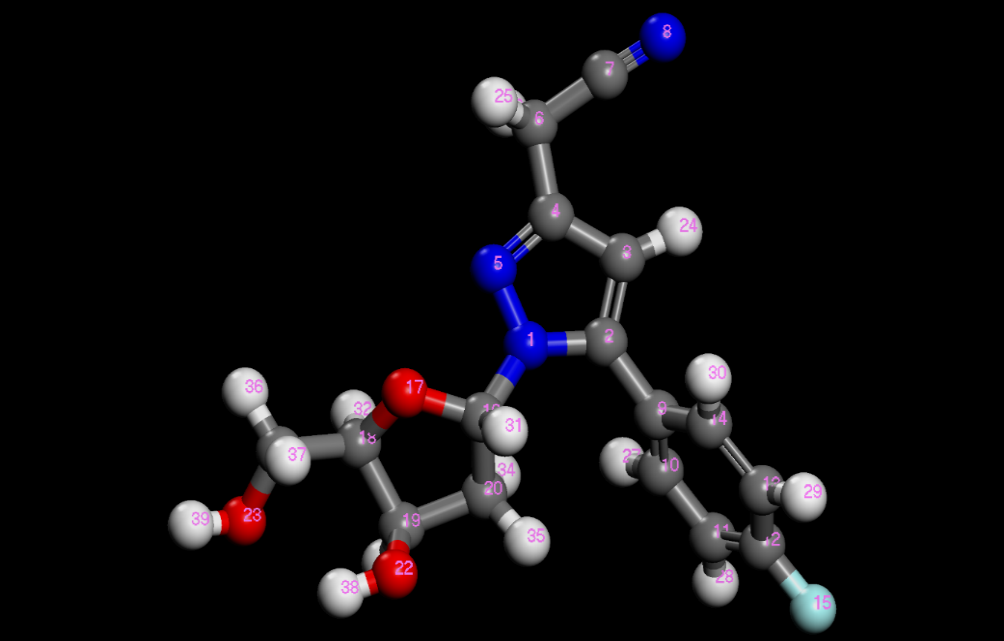** | **5d** | **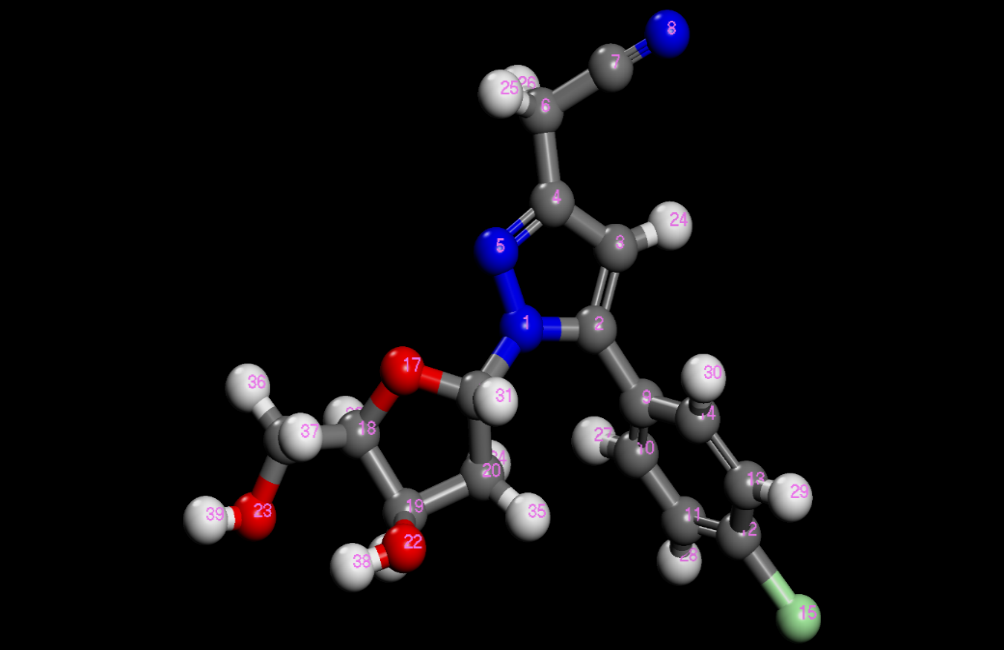** |
| **5e** | **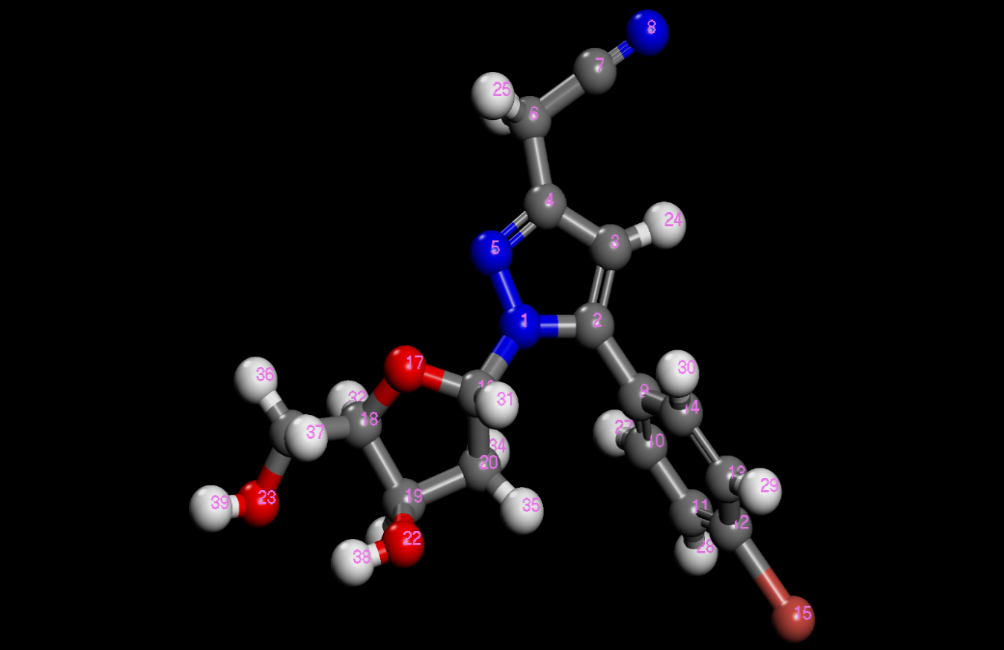** |  |  |

**Figure S1.** Alternate representation of the optimized structures of the compounds **5a-e**.

|  | Total energy | Adsorp. energy | Rigid adsorp. energy | Deform. energy | 5a dEad/dNi | Water dEad/dNi | Chloride ion dEad/dNi | Hydronium ion dEad/dNi |
| --- | --- | --- | --- | --- | --- | --- | --- | --- |
| Substrate | 0 |  |  |  |  |  |  |  |
| 5a | 92,0769 |  |  |  |  |  |  |  |
| Water | 19,41752 |  |  |  |  |  |  |  |
| Chloride ion | 0 |  |  |  |  |  |  |  |
| Hydronium ion | 21,46467 |  |  |  |  |  |  |  |
| Cu (1 1 1) (12x12x30 A vakum) - 1 | -7,24E+03 | -1,93E+04 | -7,46E+03 | -1,18E+04 | -154,399 | -28,3952 | -142,878 | -167,89 |
| Cu (1 1 1) (12x12x30 A vakum) - 2 | -7,24E+03 | -1,93E+04 | -7,46E+03 | -1,18E+04 | -138,348 | -28,7012 | -142,63 | -163,523 |
| Cu (1 1 1) (12x12x30 A vakum) - 3 | -7,24E+03 | -1,93E+04 | -7,45E+03 | -1,18E+04 | -151,61 | -28,7039 | -148,857 | -162,607 |
| Cu (1 1 1) (12x12x30 A vakum) - 4 | -7,23E+03 | -1,93E+04 | -7,46E+03 | -1,18E+04 | -138,203 | -29,3624 | -144,745 | -159,103 |
| Cu (1 1 1) (12x12x30 A vakum) - 5 | -7,23E+03 | -1,93E+04 | -7,45E+03 | -1,18E+04 | -145,581 | -28,8682 | -148,442 | -161,144 |
| Cu (1 1 1) (12x12x30 A vakum) - 6 | -7,23E+03 | -1,93E+04 | -7,45E+03 | -1,18E+04 | -146,182 | -29,396 | -147,085 | -165,613 |
| Cu (1 1 1) (12x12x30 A vakum) - 7 | -7,23E+03 | -1,93E+04 | -7,44E+03 | -1,19E+04 | -141,332 | -28,3289 | -149,132 | -160,276 |
| Cu (1 1 1) (12x12x30 A vakum) - 8 | -7,23E+03 | -1,93E+04 | -7,45E+03 | -1,18E+04 | -131,812 | -29,0746 | -142,132 | -163,169 |
| Cu (1 1 1) (12x12x30 A vakum) - 9 | -7,23E+03 | -1,93E+04 | -7,45E+03 | -1,18E+04 | -128,614 | -29,0096 | -148,538 | -164,875 |
| Cu (1 1 1) (12x12x30 A vakum) - 10 | -7,23E+03 | -1,93E+04 | -7,45E+03 | -1,18E+04 | -131,218 | -28,6659 | -143,202 | -163,225 |

**Table S1.**Outputs of adsorption energies of pyrazolylnucleosides **5a** on Cu (111) in presence of water, Chloride ion and hydronium ions.

| Structures | Total energy | Adsorp. energy | Rigid adsorp. energy | Deform. energy | 5b dEad/dNi | Water dEad/dNi | Chloride ion dEad/dNi | Hydronium ion dEad/dNi |
| --- | --- | --- | --- | --- | --- | --- | --- | --- |
| Substrate | 0 |  |  |  |  |  |  |  |
| 5b | 121,9093 |  |  |  |  |  |  |  |
| Water | 19,41752 |  |  |  |  |  |  |  |
| Chloride ion | 0 |  |  |  |  |  |  |  |
| Hydronium ion | 21,46467 |  |  |  |  |  |  |  |
| Cu (1 1 1) (12x12x30 A vakum) - 1 | -7,22E+03 | -1,93E+04 | -7,47E+03 | -1,18E+04 | -142,797 | -28,3164 | -144,827 | -160,309 |
| Cu (1 1 1) (12x12x30 A vakum) - 2 | -7,22E+03 | -1,93E+04 | -7,47E+03 | -1,18E+04 | -142,248 | -27,2403 | -144,144 | -160,497 |
| Cu (1 1 1) (12x12x30 A vakum) - 3 | -7,22E+03 | -1,93E+04 | -7,46E+03 | -1,19E+04 | -153,935 | -27,6281 | -145,202 | -167,765 |
| Cu (1 1 1) (12x12x30 A vakum) - 4 | -7,22E+03 | -1,93E+04 | -7,47E+03 | -1,18E+04 | -141,972 | -27,531 | -149,817 | -164,23 |
| Cu (1 1 1) (12x12x30 A vakum) - 5 | -7,22E+03 | -1,93E+04 | -7,48E+03 | -1,18E+04 | -162,07 | -28,6835 | -143,141 | -165,07 |
| Cu (1 1 1) (12x12x30 A vakum) - 6 | -7,22E+03 | -1,93E+04 | -7,47E+03 | -1,18E+04 | -135,267 | -29,2452 | -138,69 | -158,49 |
| Cu (1 1 1) (12x12x30 A vakum) - 7 | -7,21E+03 | -1,93E+04 | -7,47E+03 | -1,18E+04 | -152,806 | -27,7097 | -144,405 | -160,066 |
| Cu (1 1 1) (12x12x30 A vakum) - 8 | -7,21E+03 | -1,93E+04 | -7,47E+03 | -1,18E+04 | -154,948 | -29,2196 | -148,226 | -162,341 |
| Cu (1 1 1) (12x12x30 A vakum) - 9 | -7,21E+03 | -1,93E+04 | -7,46E+03 | -1,18E+04 | -154,641 | -28,5635 | -148,13 | -158,325 |
| Cu (1 1 1) (12x12x30 A vakum) - 10 | -7,21E+03 | -1,93E+04 | -7,46E+03 | -1,18E+04 | -157,228 | -28,2013 | -142,27 | -167,105 |

**Table S2.**Outputs of adsorption energies of pyrazolylnucleosides **5b** on Cu (111) in presence of water, Chloride ion and hydronium ions.

| Structures | Total energy | Adsorp. energy | Rigid adsorp. energy | Deform. energy | 5c dEad/dNi | Water dEad/dNi | Chloride ion dEad/dNi | Hydronium ion dEad/dNi |
| --- | --- | --- | --- | --- | --- | --- | --- | --- |
| Substrate | 0 |  |  |  |  |  |  |  |
| 5c | 101,7923 |  |  |  |  |  |  |  |
| Water | 19,41752 |  |  |  |  |  |  |  |
| Chloride ion | 0 |  |  |  |  |  |  |  |
| Hydronium ion | 21,46467 |  |  |  |  |  |  |  |
| Cu (1 1 1) (12x12x30 A vakum) - 1 | -7,25E+03 | -1,93E+04 | -7,48E+03 | -1,18E+04 | -147,132 | -29,2158 | -150,007 | -158,069 |
| Cu (1 1 1) (12x12x30 A vakum) - 2 | -7,25E+03 | -1,93E+04 | -7,48E+03 | -1,18E+04 | -139,838 | -27,9966 | -142,938 | -161,239 |
| Cu (1 1 1) (12x12x30 A vakum) - 3 | -7,24E+03 | -1,93E+04 | -7,46E+03 | -1,18E+04 | -140,102 | -27,9511 | -141,24 | -165,505 |
| Cu (1 1 1) (12x12x30 A vakum) - 4 | -7,23E+03 | -1,93E+04 | -7,47E+03 | -1,18E+04 | -136,65 | -29,2866 | -146,18 | -166,78 |
| Cu (1 1 1) (12x12x30 A vakum) - 5 | -7,23E+03 | -1,93E+04 | -7,46E+03 | -1,19E+04 | -143,259 | -28,0501 | -150,455 | -166,652 |
| Cu (1 1 1) (12x12x30 A vakum) - 6 | -7,23E+03 | -1,93E+04 | -7,46E+03 | -1,18E+04 | -131,198 | -28,9144 | -154,474 | -164,72 |
| Cu (1 1 1) (12x12x30 A vakum) - 7 | -7,23E+03 | -1,93E+04 | -7,46E+03 | -1,19E+04 | -166,974 | -28,6011 | -150,169 | -162,313 |
| Cu (1 1 1) (12x12x30 A vakum) - 8 | -7,23E+03 | -1,93E+04 | -7,46E+03 | -1,19E+04 | -149,521 | -29,608 | -142,267 | -156,942 |
| Cu (1 1 1) (12x12x30 A vakum) - 9 | -7,23E+03 | -1,93E+04 | -7,45E+03 | -1,19E+04 | -140,95 | -28,1092 | -142,38 | -167,214 |
| Cu (1 1 1) (12x12x30 A vakum) - 10 | -7,23E+03 | -1,93E+04 | -7,44E+03 | -1,19E+04 | -151,395 | -28,5889 | -149,305 | -167,504 |

**Table S3.**Outputs of adsorption energies of pyrazolylnucleosides **5c** on Cu (111) in presence of water, Chloride ion and hydronium ions.

| Structures | Total energy | Adsorp. energy | Rigid adsorp. energy | Deform. energy | 5d dEad/dNi | Water dEad/dNi | Chloride ion dEad/dNi | Hydronium ion dEad/dNi |
| --- | --- | --- | --- | --- | --- | --- | --- | --- |
| Substrate | 0 |  |  |  |  |  |  |  |
| 5d | 96,50193 |  |  |  |  |  |  |  |
| Water | 19,41752 |  |  |  |  |  |  |  |
| Chloride ion | 0 |  |  |  |  |  |  |  |
| Hydronium ion | 21,46467 |  |  |  |  |  |  |  |
| Cu (1 1 1) (12x12x30 A vakum) - 1 | -7,26E+03 | -1,93E+04 | -7,48E+03 | -1,18E+04 | -137,423 | -28,3702 | -137,522 | -169,292 |
| Cu (1 1 1) (12x12x30 A vakum) - 2 | -7,24E+03 | -1,93E+04 | -7,47E+03 | -1,18E+04 | -132,434 | -28,5046 | -149,887 | -163,332 |
| Cu (1 1 1) (12x12x30 A vakum) - 3 | -7,24E+03 | -1,93E+04 | -7,46E+03 | -1,18E+04 | -137,201 | -28,9713 | -144,793 | -160,413 |
| Cu (1 1 1) (12x12x30 A vakum) - 4 | -7,24E+03 | -1,93E+04 | -7,46E+03 | -1,18E+04 | -144,938 | -28,7424 | -141,974 | -162,897 |
| Cu (1 1 1) (12x12x30 A vakum) - 5 | -7,24E+03 | -1,93E+04 | -7,46E+03 | -1,18E+04 | -137,612 | -29,2397 | -140,757 | -164,628 |
| Cu (1 1 1) (12x12x30 A vakum) - 6 | -7,23E+03 | -1,93E+04 | -7,45E+03 | -1,18E+04 | -134,03 | -28,6541 | -151,861 | -163,494 |
| Cu (1 1 1) (12x12x30 A vakum) - 7 | -7,23E+03 | -1,93E+04 | -7,46E+03 | -1,18E+04 | -145,707 | -28,4822 | -144,427 | -163,049 |
| Cu (1 1 1) (12x12x30 A vakum) - 8 | -7,23E+03 | -1,93E+04 | -7,46E+03 | -1,18E+04 | -137,835 | -27,5052 | -141,391 | -164,997 |
| Cu (1 1 1) (12x12x30 A vakum) - 9 | -7,23E+03 | -1,93E+04 | -7,45E+03 | -1,18E+04 | -138,108 | -28,6774 | -151,79 | -158,13 |
| Cu (1 1 1) (12x12x30 A vakum) - 10 | -7,23E+03 | -1,93E+04 | -7,45E+03 | -1,18E+04 | -132,374 | -27,6292 | -144,098 | -163,8 |

**Table S4.**Outputs of adsorption energies of pyrazolylnucleosides **5d** on Cu (111) in presence of water, Chloride ion and hydronium ions.

| Structures | Total energy | Adsorp. energy | Rigid adsorp. energy | Deform. energy | 5e dEad/dNi | Water dEad/dNi | Chloride ion dEad/dNi | Hydronium ion dEad/dNi |
| --- | --- | --- | --- | --- | --- | --- | --- | --- |
| Substrate | 0 |  |  |  |  |  |  |  |
| 5e | 382,0772 |  |  |  |  |  |  |  |
| Water | 19,41752 |  |  |  |  |  |  |  |
| Chloride ion | 0 |  |  |  |  |  |  |  |
| Hydronium ion | 21,46467 |  |  |  |  |  |  |  |
| Cu (1 1 1) (12x12x30 A vakum) - 1 | -7,27E+03 | -1,96E+04 | -7,50E+03 | -1,21E+04 | -441,2230792 | -28,43711797 | -144,5366082 | -161,8317252 |
| Cu (1 1 1) (12x12x30 A vakum) - 2 | -7,25E+03 | -1,96E+04 | -7,47E+03 | -1,21E+04 | -448,2158508 | -26,88625171 | -139,6464266 | -161,5959164 |
| Cu (1 1 1) (12x12x30 A vakum) - 3 | -7,25E+03 | -1,96E+04 | -7,47E+03 | -1,21E+04 | -438,3916324 | -28,89967747 | -151,7714056 | -158,2372643 |
| Cu (1 1 1) (12x12x30 A vakum) - 4 | -7,25E+03 | -1,96E+04 | -7,47E+03 | -1,21E+04 | -431,5122849 | -27,62954311 | -149,617233 | -167,8856879 |
| Cu (1 1 1) (12x12x30 A vakum) - 5 | -7,25E+03 | -1,96E+04 | -7,48E+03 | -1,21E+04 | -436,2708233 | -28,34861321 | -146,9496491 | -161,2288137 |
| Cu (1 1 1) (12x12x30 A vakum) - 6 | -7,25E+03 | -1,96E+04 | -7,48E+03 | -1,21E+04 | -448,2183595 | -27,89760095 | -148,2266055 | -164,4711769 |
| Cu (1 1 1) (12x12x30 A vakum) - 7 | -7,24E+03 | -1,96E+04 | -7,46E+03 | -1,21E+04 | -435,8871605 | -28,86242859 | -148,694876 | -162,9978881 |
| Cu (1 1 1) (12x12x30 A vakum) - 8 | -7,24E+03 | -1,96E+04 | -7,46E+03 | -1,21E+04 | -435,6602402 | -28,78094125 | -142,8027965 | -156,0802244 |
| Cu (1 1 1) (12x12x30 A vakum) - 9 | -7,24E+03 | -1,96E+04 | -7,46E+03 | -1,21E+04 | -433,0099968 | -28,5376723 | -147,4164001 | -163,0553721 |
| Cu (1 1 1) (12x12x30 A vakum) - 10 | -7,24E+03 | -1,96E+04 | -7,47E+03 | -1,21E+04 | -439,8257128 | -28,58914622 | -143,693473 | -161,4771867 |

**Table S5.**Outputs of adsorption energies of pyrazolylnucleosides **5e** on Cu (111) in presence of water, Chloride ion and hydronium ions.
